# Morphological changes of plasma membrane and protein assembly during clathrin-mediated endocytosis

**DOI:** 10.1371/journal.pbio.2004786

**Published:** 2018-05-03

**Authors:** Aiko Yoshida, Nobuaki Sakai, Yoshitsugu Uekusa, Yuka Imaoka, Yoshitsuna Itagaki, Yuki Suzuki, Shige H. Yoshimura

**Affiliations:** 1 Graduate School of Biostudies, Kyoto University, Kyoto, Japan; 2 R&D Group, Olympus Corporation, Hachioji, Japan; 3 Frontier Research Institute for Interdisciplinary Sciences, Tohoku University, Sendai, Japan; UT Southwestern Medical Center, United States of America

## Abstract

Clathrin-mediated endocytosis (CME) proceeds through a series of morphological changes of the plasma membrane induced by a number of protein components. Although the spatiotemporal assembly of these proteins has been elucidated by fluorescence-based techniques, the protein-induced morphological changes of the plasma membrane have not been fully clarified in living cells. Here, we visualize membrane morphology together with protein localizations during CME by utilizing high-speed atomic force microscopy (HS-AFM) combined with a confocal laser scanning unit. The plasma membrane starts to invaginate approximately 30 s after clathrin starts to assemble, and the aperture diameter increases as clathrin accumulates. Actin rapidly accumulates around the pit and induces a small membrane swelling, which, within 30 s, rapidly covers the pit irreversibly. Inhibition of actin turnover abolishes the swelling and induces a reversible open–close motion of the pit, indicating that actin dynamics are necessary for efficient and irreversible pit closure at the end of CME.

## Author summary

Cells communicate with their environments via the plasma membrane and various membrane proteins. Clathrin-mediated endocytosis (CME) plays a central role in such communication and proceeds with a series of multiprotein assembly, deformation of the plasma membrane, and production of a membrane vesicle that delivers extracellular signaling molecules into the cytoplasm. In this study, we utilized our home-built correlative imaging system comprising high-speed atomic force microscopy (HS-AFM) and confocal fluorescence microscopy to simultaneously image morphological changes of the plasma membrane and protein localization during CME in a living cell. The results revealed a tight correlation between the size of the pit and the amount of clathrin assembled. Actin dynamics play multiple roles in the assembly, maturation, and closing phases of the process, and affects membrane morphology, suggesting a close relationship between endocytosis and dynamic events at the cell cortex. Knock down of dynamin also affected the closing motion of the pit and showed functional correlation with actin.

## Introduction

Cells communicate with the extracellular environment via the plasma membrane and membrane proteins. They transduce extracellular signals and substances into the cellular plasma via cell surface receptors, channels, and pumps, as well as by various endocytic processes [1–3]. Cells also disseminate their intracellular contents to the extracellular space via exocytosis. These dynamic cellular processes are largely dependent on the assembly and catalytic function of various proteins in the plasma membrane. Clathrin-mediated endocytosis (CME) is conducted by more than 30 different proteins. Extensive studies using fluorescence imaging techniques revealed the spatiotemporal dynamics of individual proteins in living cells [4–6]. In addition, a number of in vitro studies revealed unique functions of these proteins in deforming the plasma membrane [7]. For instance, Bin-amphiphysin-Rvs17 (BAR) domain proteins bind to the surface of the lipid bilayer and induce membrane curvature and tubulation and are therefore presumed to be involved in membrane deformation in an early stage of CME [8]. Dynamin also induces membrane tubulation with a smaller diameter and vesiculation via a nucleotide-dependent conformational change, and therefore has been considered to be involved in the vesicle scission process [9,10].

Despite our increasingly detailed knowledge regarding the cellular dynamics of these proteins in vivo and their catalytic activity in vitro, the morphological changes of the plasma membrane during CME in living cells have not been studied. This has mainly been due to a lack of imaging techniques for visualizing the membrane. Electron microscopy (EM) has made a substantial contribution to the study of CME, owing to its high spatial resolution. The detailed morphological changes of the plasma membrane, together with the assembly of proteins, such as clathrin, have been imaged and analyzed in a series of images to understand the entire process of CME [11–15]. However, aligning a thousand EM snapshots still suffers from a large limitation in the time resolution. In contrast to EM, fluorescence labeling and imaging techniques are powerful tools for studying protein dynamics in living cells. Recent advances in these techniques allow time-lapse imaging of a single protein molecule in a living cell with subsecond time resolution. However, it is not suitable for imaging morphological changes of the plasma membrane in a living cell at a submicrometer scale.

Scanning probe microscopies, including atomic force microscopy (AFM), are powerful approaches for characterizing the surface of a specimen at nanometer resolution. Notably, high-speed AFM (HS-AFM) has been utilized to visualize various molecular structures and reactions at subsecond resolution in vitro [16–19]. We recently developed an HS-AFM for live-cell imaging and successfully visualized structural dynamics of the plasma membrane in living cells [20,21]. In this study, we utilize this HS-AFM to analyze the morphological changes of the plasma membrane during CME. To understand the role of specific proteins during the morphological change, HS-AFM is combined with confocal laser scanning microscopy (CLSM) so that we could simultaneously visualize membrane structures and protein localizations during CME in living cells. Overlaying AFM and fluorescence images reveals the dynamics of protein assembly and concomitant morphological changes of the plasma membrane with high spatial resolution. In particular, we elucidate the role of actin in the closing step of CME.

## Results

### Hybrid imaging with combined HS-AFM and CLSM

**Fig 1:**
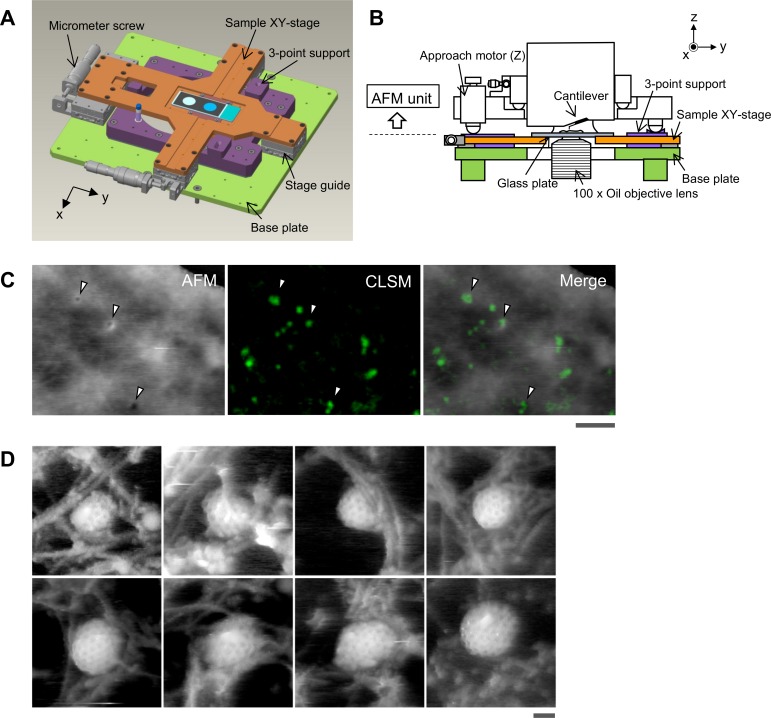
Aligning the confocal image and the AFM image. (A) Schematic illustration of the sample stage. A cross-shaped movable XY-stage (orange) is mounted on the base plate (light green) of the inverted optical microscope (IX83) via a stage guide (gray) equipped at each of the 4 ends of the cross. A 3-point support plate (purple) for mounting the AFM scanner unit is fixed on the base plate with a configuration that does not hinder the sliding of the XY-stage along the x-axis and y-axis. These setups allow the sample stage to move independently of the AFM unit and the optical axis. (B) Side view of the HS-AFM unit mounted on the stage illustrated in panel A. (C) Overlaying a confocal image and an AFM image. COS-7 cells expressing EGFP-CLCa were fixed with 5% paraformaldehyde and subjected to AFM (left) and CLSM (middle) imaging. The x-y position of the probe tip was determined as described in S1 Fig. Two images were overlaid (right) based on the x-y center position. Scale bar: 1 μm. Autofluorescence of the probe was much weaker than clathrin spot and could not be detected during the fast scanning. (D) AFM images of CCP on the cytoplasmic surface of the plasma membrane. COS-7 cells were “unroofed” by mild sonication as described in Materials and methods and then fixed with glutaraldehyde. Scale bar: 0.1 μm. AFM, atomic force microscopy; CCP, clathrin-coated pit; CLSM, confocal laser scanning microscopy; COS-7, CV-1 in origin with SV40 gene line 7; EGFP, enhanced green fluorescent protein; EGFP-CLCa, EGFP-fused clathrin light chain a; HS-AFM, high-speed AFM.

To reveal protein-induced membrane deformation during CME in a living cell, we first established a hybrid imaging system with HS-AFM and CLSM. We previously reported the development of a tip-scanning AFM unit and its combination with an inverted optical microscope with a fluorescence illumination unit [20,21]. In this study, we combined the HS-AFM unit with an inverted optical microscope equipped with a confocal laser scanning unit to increase the optical resolution. To obtain stable imaging, the stage was redesigned. The detailed configuration of the stage is described in Fig 1A and 1B. A cross-shaped movable XY-stage is mounted on the base plate of the inverted optical microscope stage, which allows the specimen to move independently of the AFM unit and the objective lens. The AFM scanning unit now has a 6.0 × 4.5 μm^2^ scanning area, which is larger than the previous unit (4.0 × 3.0 μm^2^), to enable imaging of a larger area of the cell surface.

We then established a procedure for aligning confocal fluorescence and AFM images. The details of the alignment method are described in S1 Fig. In brief, the probe was brought to approach and attach on the glass surface without scanning. The z-position of the probe tip and confocal plane was also aligned by setting the glass surface as a reference position (z = 0). The x-y position of the probe tip on the optical axis was determined by imaging an autofluorescence signal of the probe (S1A and S1B Fig). The center position of the probe fluorescence was defined as an origin (x, y = 0, 0), and the x-y position of the AFM image also refers to this scale (S1B Fig). Once the reference position of the probe tip and optical axis was determined, the cross-shaped sample stage allowed the specimen (cell) to move in x-y directions without changing the relative position between the HS-AFM and CLSM.

The spatial accuracy of the hybrid imaging described above was tested by observing a chemically fixed cell. CV-1 in origin with SV40 gene line 7 (COS-7) cells expressing enhanced green fluorescent protein (EGFP)-fused clathrin light chain a (EGFP-CLCa) were fixed and observed by hybrid imaging. Several different membrane structures could be identified in the AFM image of the cell surface (Fig 1): membrane invaginations of different sizes (diameters); ruffle-like short narrow protrusions; and large swellings. When the AFM image was overlaid on the confocal fluorescence image of EGFP-CLCa based on the x-y position alignment, several clathrin spots readily colocalized with membrane invaginations identified in the AFM image (Fig 1C). The section profile analysis revealed that the diameter of the membrane invaginations (pits) identified in the AFM images ranged between 150 and 400 nm, whereas those colocalized with a clathrin spot ranged between 150 and 350 nm (237 ± 78 nm [mean ± SD]; *n* = 4) (the details of the AFM image analysis are provided in S2 Fig). Because this size range of clathrin-coated pits (CCPs) was larger than the previously reported ones (20–175 nm) [11], we examined the size of the entire CCP by observing the cytoplasmic side of the plasma membrane. COS-7 cells were unroofed by the procedure previously described [22], fixed, and then observed by HS-AFM. As shown in Fig 1D, CCPs with hexagonal network of clathrin were clearly observed. The diameter ranged between 150 and 400 nm (225 ± 49 nm [mean ± SD]; *n* = 45), which approximately corresponds to 130 to 380 nm after subtracting the tip curvature. Therefore, we concluded that the membrane invaginations with clathrin fluorescence spot observed by our hybrid imaging system were CCPs, and the size of the pit ranged from 150 to 400 nm. This result matches well to previous EM observations of the CCP in COS-7 cells, in which CCPs larger than 150 nm were often observed [23].

To evaluate the accuracy of the image alignment, the position (x, y) of the membrane invagination in the AFM image was compared with that of the corresponding fluorescence spot in the confocal image. The x-y offset between the centroid of the membrane invagination in the AFM image and the fluorescent spot of clathrin was 27 ± 20 nm (mean ± SD; *n* = 7). Considering the diameter of the membrane invaginations (150–400 nm), we concluded that our image-overlaying procedure is accurate enough to merge the clathrin spot with the AFM image. It should be noted that the number of membrane invaginations in the AFM image was less than that of the fluorescent clathrin spots—i.e., there were some clathrin spots that did not colocalize with membrane invaginations in the AFM image. Such clathrin signals could be due to either clathrin-coated vesicles (CCVs) that had already budded from the plasma membrane or CCPs that formed in the basal surface of the cell.

### Live-cell hybrid imaging of membrane morphology and protein assembly in CME

**Fig 2:**
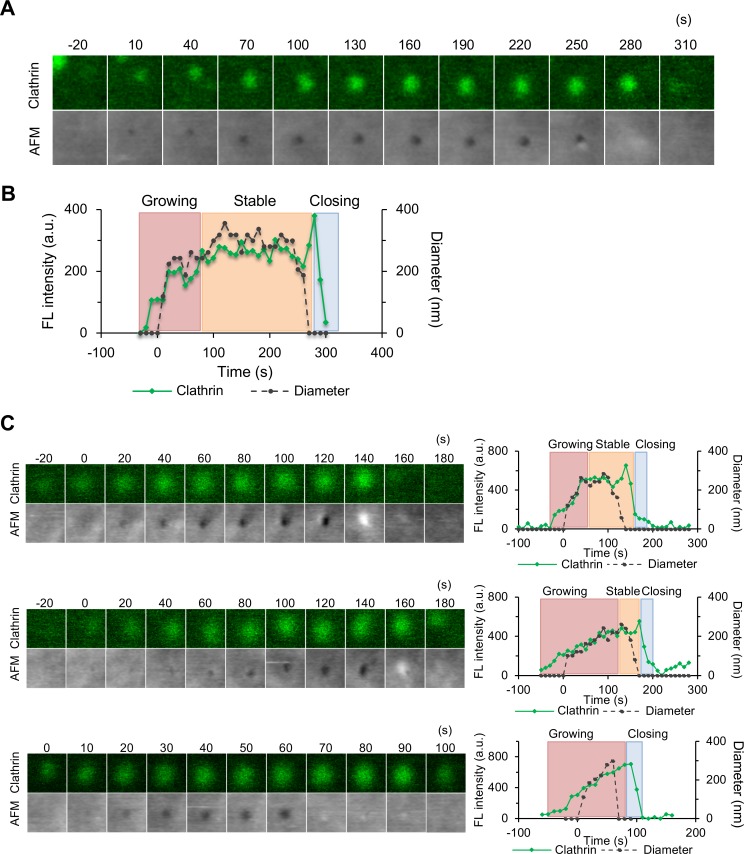
Hybrid AFM imaging reveals morphological changes of CCPs in living cells. In culture medium, live COS-7 cells expressing EGFP-CLCa were subjected to time-lapse hybrid AFM imaging. The position of the probe tip was aligned as described in S1 Fig. (A) The fluorescence image (top) and AFM image (bottom) of a representative CCP. Image size: 0.6 × 0.6 μm^2^. (B) The fluorescence intensity (green) and diameter of the CCPs in the AFM images (dotted black) in panel A are plotted against time. Based on the changes of the clathrin signal, the whole process is divided into a growing phase, a stable phase, and a closing phase based on the procedure described in S3 Fig. (C) Variations of CCPs observed by hybrid time-lapse imaging. Individual CCPs have large variations in the duration of the growing and stable phases. Underlying data may be found in S1 Data. AFM, atomic force microscopy; a.u., arbitrary unit; CCP, clathrin-coated pit; COS-7, CV-1 in origin with SV40 gene line 7; EGFP, enhanced green fluorescent protein; EGFP-CLCa, EGFP-fused clathrin light chain a; FL, fluorescence.

To follow the entire process of CME, we then performed time-lapse hybrid imaging of living cells. A live COS-7 cell expressing EGFP-CLCa was observed by HS-AFM and CLSM in culture medium containing fetal bovine serum (FBS). The temperature was kept constant at 28 °C, at which the entire CME process—including scission step—was demonstrated to occur [24]. Both AFM and CLSM images were obtained every 10 s, and overlaid. Small membrane invaginations as described in Fig 1 were colocalized with EGFP-CLCa spots over several minutes (Fig 2A and 2B) (see video in S1 Movie). The average x-y offset of the fluorescent spot from the corresponding membrane pit in the AFM image was 38 ± 13 nm (mean ± SD; *n* = 8) (S3 Fig), which was slightly larger than that in the fixed cell (27 ± 20 nm). This is partly due to the time lag between AFM and CLSM scanning; although AFM and CLSM images were taken with the same frame rate (10 s/frame), the exact timing of data recording at a certain position in the scanning area was not perfectly synchronized because 2 different scanning systems are operated by 2 independent controllers. Considering the fact that CCPs are diffusing on the membrane (S1 Movie), the time lag between 2 scanning systems results in a small offset of each CCP spot between 2 different images. However, because this offset is much smaller than the size of the CCP (150–400 nm), we concluded that our hybrid time-lapse imaging procedure has spatial resolution high enough for assigning individual clathrin spots to the membrane invaginations in the AFM image.

Because the plasma membrane of a living cell is always fluctuating in x, y, and z directions, we carefully examined the effect of scanning parameter on the morphologies of CCPs. When the amplitude of the cantilever was increased by 5%, cortical actin network beneath the plasma membrane became more visible (see S4A Fig), which sometimes occurred during our time-lapse observation. In such a condition, the CCP (an arrow in S4A Fig) was still clearly observed, and the difference in the diameter was less than 9%, indicating that this amount of fluctuation had little effect on the morphological analyses of the CCP during CME (see later sections). In addition, “tip skipping” sometimes occurred near the CCP (S4B Fig). However, the frequency of tip skipping was very low (12 out of 272 CCPs), and tip skipping occurred on 1 or 2 consecutive scanning lines, which corresponds to approximately 37 nm. From these analyses, we concluded that fluctuation of tip–sample interaction does not affect the morphological analyses of the CCPs.

The fluorescence intensity of the clathrin spot and the diameter of the pit in the AFM image were plotted against time (Fig 2B). The clathrin signal appeared 20 to 30 s before the membrane started to deform. The clathrin signal then increased (growing phase) until it reached a stable phase as demonstrated by a previous study [25] (see S5A Fig for the definition of individual phases). During the growing phase, the aperture of the pit in the AFM images also increased (Fig 2B), suggesting that the size of the pit also enlarged during this period. During the stable phase, the aperture also remained almost constant. There were large variations in the duration of the growing and stable phases; the growing phase ranged between 40 and 280 s, and the subsequent stable phase lasted between 0 and 260 s (*n* = 35) (Fig 2C). Following the stable phase, the closing phase proceeded over a short period of time (20–50 s) (Fig 2B and 2C). Some pits closed in a single frame (<10 s). When these fast-closing events were observed at a higher scanning rate (1 s/frame), the pit closed in as fast as 3 s (S5B Fig). Notably, the clathrin spot remained for another 20 to 30 s after the pit closed and then suddenly disappeared. This could indicate that either the clathrin coat remains on the vesicle and is eventually disassembled by G-associated kinase (GAK) [26,27], or the vesicle eventually moves out of the focal plane of the CLSM. We obtained a similar result when the pit area, instead of the pit diameter, was plotted against time (S5C Fig). The total lifetime of the CCP ranged from 40 to 330 s (*n* = 113).

### Assembly of other CCP-related proteins

**Fig 3:**
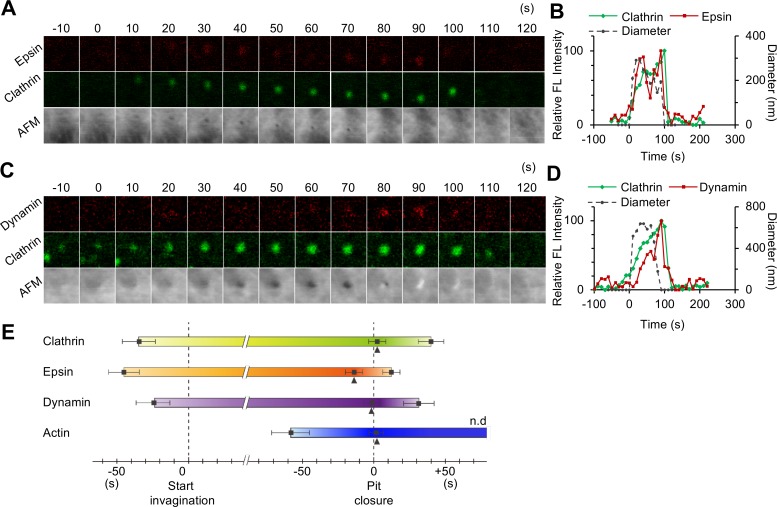
Morphological changes of the plasma membrane and protein assembly during CME. (A, B) Time-lapse hybrid imaging of COS-7 cells expressing EGFP-CLCa and mCherry-epsin. Fluorescence images for epsin and clathrin, as well as AFM images are shown every 10 s (panel A). Image size: 1.2 × 1.2 μm^2^. The signal intensities of the fluorescence spots for clathrin (green) and epsin (red), and the diameter of the membrane invagination in the AFM image (dotted black) were plotted against time (panel B). The FL intensity is plotted relative to its maximum value. (C, D) Time-lapse hybrid imaging of COS-7 cells expressing EGFP-CLCa and Dyn2-mCherry. Fluorescence images for dynamin and clathrin, as well as AFM images are shown every 10 s (panel C). The signal intensities of the fluorescence spots for clathrin (green) and dynamin (red), and the diameter of the membrane invagination in the AFM image (dotted black), were plotted against time (panel D). The FL intensity is plotted relative to its maximum value. (E) A summary of protein assembly at CCPs. Based on the AFM images, the time when the plasma membrane started to invaginate (left half) and when the pit had completely closed (right half) were defined as t = 0. Arrowheads indicate the time when fluorescence signal was maximum. The results of hybrid imaging for clathrin, epsin, dynamin, and actin are summarized along this time scale. The timings when the fluorescence signal appeared on the CCP (left half) and disappeared (right half) are plotted. *n* = 35 for clathrin, *n* = 8 for epsin, *n* = 13 for dynamin, and *n* = 14 for actin. Error bars represent SD. Underlying data may be found in S1 Data. AFM, atomic force microscopy; CCP, clathrin-coated pit; CME, clathrin-mediated endocytosis; COS-7, CV-1 in origin with SV40 gene line 7; EGFP, enhanced green fluorescent protein; EGFP-CLCa, EGFP-fused clathrin light chain a; FL, fluorescence; n.d., not determined.

Assemblies of other CCP-related proteins were also investigated by time-lapse hybrid imaging. Epsin is known to add bending stress to the lipid bilayer at an early stage of CME, thus changing the membrane curvature [28], and it recruits clathrin to the pit surface. COS-7 cells simultaneously expressing mCherry-fused epsin and EGFP-CLCa were subjected to time-lapse hybrid imaging. Epsin started to assemble on the plasma membrane prior to the membrane invagination, which was similar to clathrin (Fig 3A and 3B; S2 Movie) (see also S6 Fig for other observations). However, statistical analysis of the timing of assembly and membrane invagination revealed that epsin assembles prior to clathrin; fluorescence spots of epsin and clathrin appeared 47 ± 9 (mean ± SD; *n* = 8) and 34 ± 13 s (mean ± SD; *n* = 35), respectively, before the membrane started to invaginate (Fig 3E). During the growing phase, fluorescence signals of both proteins increased (Fig 3B). Epsin and clathrin signals peaked at 14 ± 5 s (*n* = 8) before and 3 ± 7 s (*n* = 35) after the pit closed, and they disappeared at 13 ± 5 s (*n* = 8) and 39 ± 13 s (*n* = 35) after the closure, respectively. All of these results demonstrated that CCPs in COS-7 cells show a large variation in the lifetime (40–280 s), and their opening and closing events are tightly coupled with protein assembly.

Dynamin localizes at the neck of the pit and plays a role in the vesicle scission [15] in the last step of CME. COS-7 cells expressing mCherry-fused dynamin 2 (an isoform ubiquitously expressed in a variety of cells) together with EGFP-CLCa were subjected to the time-lapse hybrid imaging. Although dynamin plays a role in the vesicle scission, a dynamin signal started to assemble on the CCP in an early stage of CME as demonstrated in previous studies [4,29]; in our observations, it assembled 25 ± 12 s (mean ± SD; *n* = 13) before the membrane invagination began (Fig 3C, 3D and 3E, S3 Movie) (see S7 Fig for other observations). The signal gradually increased in the growing phase, but during the stable phase, the dynamin signal was not clearly defined compared to clathrin. The dynamin signal peaked with the same timing as pit closure and gradually decreased thereafter (it completely disappeared 38 ± 9 s [mean ± SD; *n* = 13] after the closure), consistent with the notion that it is involved in the last step of CME.

**Fig 4:**
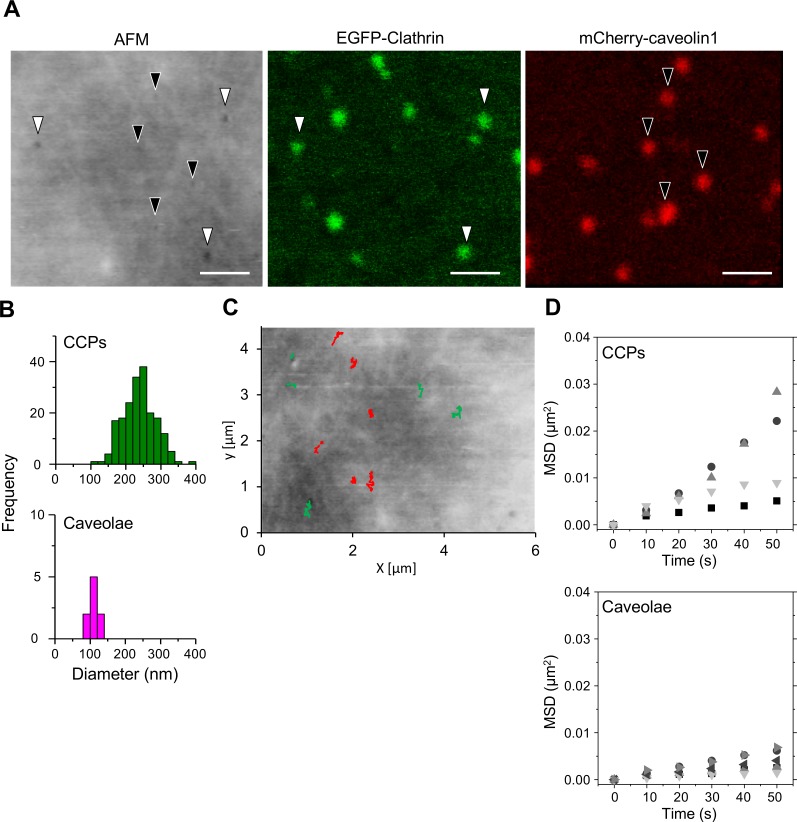
Hybrid AFM imaging distinguishes CCPs from caveolae in live cells. COS-7 cells expressing both EGFP-CLCa and mCherry-caveolin1 in culture medium were subjected to hybrid time-lapse AFM imaging. (A) The x-y alignment of the obtained AFM image (left), EGFP image (middle), and mCherry image (right) was performed as described in S1 Fig. Small membrane invaginations that colocalized with an EGFP signal or with an mCherry signal are indicated by white and black arrowheads, respectively. Scale bars: 1 μm. (B) The diameters of the membrane invaginations that colocalized with a clathrin signal (top panel) or with a caveolin signal (bottom panel) were measured by section analysis and are summarized as a histogram. (C, D) Lateral movements of CCPs and caveolae were analyzed. The trajectories of the pit center (green for clathrin, red for caveolae) were superimposed on the AFM image (panel C), and the MSD was plotted against time (panel D). Top panel: CCP; bottom panel: caveolae. A lateral drift of the specimen was estimated by measuring the average displacement of all pits between the initial and the last frame of the analysis. Because the average displacement of randomly diffusing spots is zero (|*d*| = 0), the average displacement of all endocytic pits observed in a series of AFM images corresponds to the drift of specimen during the observation. Underlying data may be found in S1 Data. AFM, atomic force microscopy; CCP, clathrin-coated pit; COS-7, CV-1 in origin with SV40 gene line 7; EGFP, enhanced green fluorescent protein; EGFP-CLCa, EGFP-fused clathrin light chain a; MSD, mean square displacement.

We further confirmed that the membrane pits observed were indeed CCPs and not other types of endocytic structures. Caveolae are found in another endocytic pathway that is mediated by other sets of proteins (caveolin, etc.) but also includes invagination of the plasma membrane. mCherry-fused caveolin1, a major component of caveolae, was expressed in COS-7 cells together with EGFP-CLCa, and live cells were subjected to time-lapse hybrid imaging. The clathrin spots did not colocalize with caveolin1 spots during the observation. Overlaying 3 images (AFM, EGFP-CLCa, and mCherry-caveolin1) clearly revealed morphological differences between CCP and caveolae (Fig 4A, see also S4 Movie). The aperture of caveolae ranged from 80 to 120 nm, whereas that of CCPs ranged from 150 to 400 nm (Fig 4B). Similar to the CCP, the aperture of the caveolae observed by AFM was slightly larger than that observed by EM [30], probably for the same reason as described above. Caveolae had longer lifetimes than CCPs; the average lifetime of a CCP was 81 ± 55 s, whereas caveolae remained open for over 400 s. They also showed different lateral movements in the plasma membrane; the diffusion coefficient of CCPs was 7.3 × 10^−9^ (cm^2^ s^−1^), whereas that of caveolae was 2.1 × 10^−9^ (cm^2^ s^−1^) (Fig 4C and 4D). Taken together, these results indicate that time-lapse hybrid imaging could identify and distinguish the different aperture openings and diffusion kinetics of the 2 types of invaginations.

### Unique morphologies of the plasma membrane during CME

**Fig 5:**
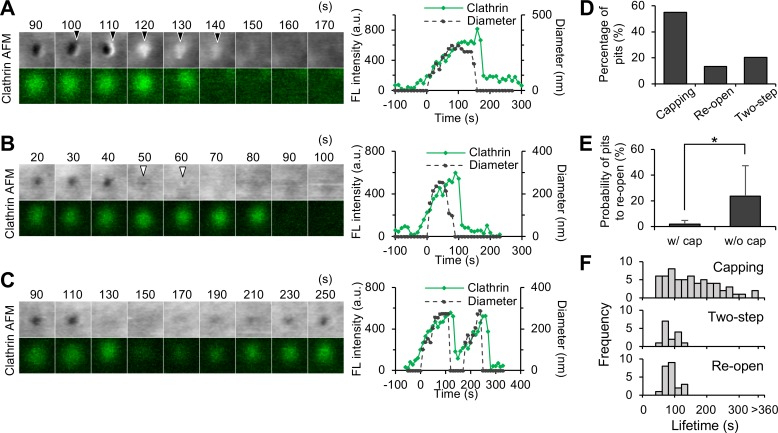
Variations in the closing motions of CCPs. Membrane morphologies of CCPs when they close were analyzed from the same data set shown in Fig 3A, 3B and 3C. Three different motions identified in the CCPs: capping (panel A), two-step (panel B), and re-opening (panel C). AFM images and fluorescence images of EGFP-CLCa are shown in the panels at left. Fluorescence intensity (green) and the diameter of the CCP (dotted black) are also plotted in the time course (right panels). Image size: 0.6 × 0.6 μm^2^. Membrane swelling in the capping motion, and the small aperture in the two-step motion, are indicated by black and white arrowheads, respectively. (D) The frequency of each closing motion. In total, 113 CCPs were analyzed. The frequency of 3 closing motions (capping, re-open, and two-step) were counted and represented as a percentage. The pits without any of these motions were not counted here. Three motions were not mutually exclusive, and sometimes multiple motions were seen in the identical pits. (E) Relationship between capping motion and re-opening motion. Among the CCPs that showed re-opening motion, the ratios of the CCPs with or without a capping motion are plotted. Error bars represent SD. (F) Distribution of the CCP lifetime for 3 different closing motions. Underlying data may be found in S1 Data. AFM, atomic force microscopy; a.u., arbitrary unit; CCP, clathrin-coated pit; EGFP, enhanced green fluorescent protein; EGFP-CLCa, EGFP-fused clathrin light chain a; FL, fluorescence.

In contrast to the growing and stable phases, which continue for more than 1 min, the closing and disassembly phase was completed relatively rapidly (<30 s). In many cases, the membrane aperture suddenly disappeared (Fig 2). However, the detailed image analyses revealed several unique membrane structures and dynamics in the closing step of the CCP, which include (i) capping, (ii) two-step, and (iii) re-opening (Fig 5A–5C). The capping motion was frequently observed in more than 50% of the CME events (54.9%, Fig 5D, S1 Table). A small membrane region adjacent to the CCP swelled and eventually covered over the pit (Fig 5A). The section profile analysis (S2 Fig) revealed that the swelling region was 378 ± 62 nm in diameter and 38 ± 10 nm in height (mean ± SD; *n* = 13), which is comparable to the pit size. The entire closing motion took 23 ± 13 s (mean ± SD; *n* = 48). This structure is very similar to the membrane protrusion observed by ion-conductance microscopy [31] (see Discussion section for details).

Two-step closing was observed in approximately 20% of the CME events (Fig 5D). The pit aperture first decreased to approximately 120 nm and then disappeared (Fig 5B). The duration of the small-aperture step was <40 s. AFM imaging with higher time resolution (2 s/frame) revealed two-step motions with faster small-aperture steps (approximately 10 s). These results indicate that many CCPs close with a two-step motion, but the duration of the smaller-aperture step varied from several seconds to 40 s. A re-opening motion was also observed in more than 10% of the total CME (Fig 5D). A pit once closed completely then re-opened after several frames of the closure (Fig 5C). The duration of the closed state varied between 10 and 70 s (30 ± 24 s, *n* = 8). During the closed state, clathrin signal decreased to between 14% and 70% (39 ± 18%, *n* = 8) and re-increased after the pit re-opened (Fig 5C). The position of re-opening was within 13 to 113 nm (52 ± 31 nm, *n* = 8) from the closed position. These observations were similar to what was previously described as “hot spot,” in which multiple cycles of assembly and disassembly of endocytic proteins such as clathrin or dynamin occurred in a limited area of membrane [32–34] (see Discussion section for details).

There was a clear distinction between the capping and re-opening motions: pits that closed with capping did not tend to re-open (Fig 5E), suggesting that capping plays a role in irreversible closing. In contrast, the two-step motion was not mutually exclusive to other motions so that we sometimes observed a two-step motion that finally culminated with capping (S8 Fig). The comparison of the total lifetime revealed a wide distribution in capping-ended pits, whereas two-step and re-opening motions showed a narrow distribution of about 100 s (Fig 5F).

### Involvement of actin turnover in the closing step

**Fig 6:**
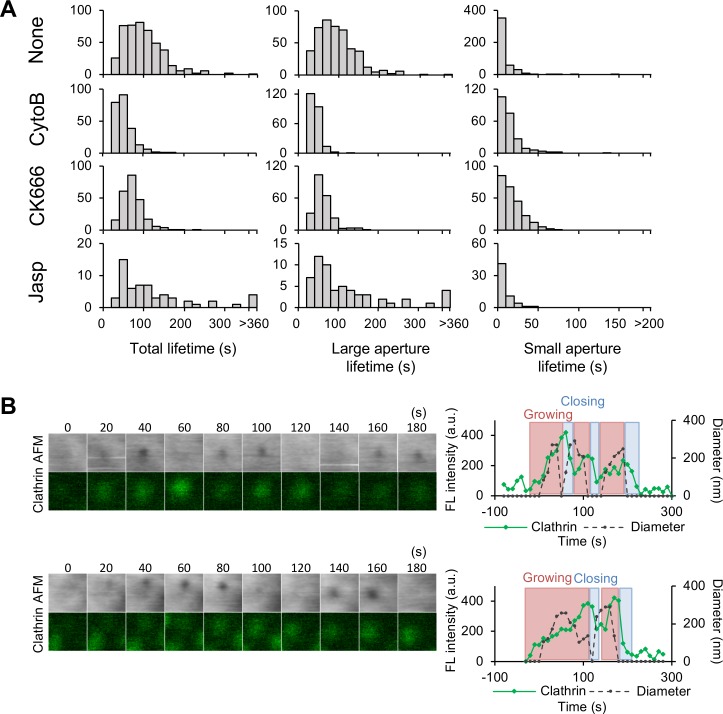
Effects of actin-related inhibitors on CCP lifetime. (A) COS-7 cells on the microscope stage were treated with cytochalasin B, jasplakinolide, or CK666 and were subjected to time-lapse hybrid imaging. The total lifetime and durations of large and small apertures in the two-step motion were measured and summarized as histograms. (B) Time-lapse AFM and fluorescence images obtained from COS-7 cells expressing EGFP-CLCa after treatment with cytoB (left panels). Image size: 0.6 × 0.6 μm^2^. The fluorescence intensity (green) and the diameter of the CCPs in the AFM image (dotted black) are plotted against time (right panels). The process is divided into growing (red), stable (orange), and closing (blue) phases as described in Fig 2. Underlying data may be found in S1 Data. AFM, atomic force microscopy; a.u., arbitrary unit; CCP, clathrin-coated pit; COS-7, CV-1 in origin with SV40 gene line 7; cytoB, cytochalasin B; EGFP, enhanced green fluorescent protein; EGFP-CLCa, EGFP-fused clathrin light chain a; FL, fluorescence; jasp, jasplakinolide.

Actin and actin-related proteins are also known to contribute to CCP assembly, although their exact role is not fully understood [14,35]. We previously observed and reported the dynamic turnover of the cortical actin network [36]; actin filaments are polymerized near the plasma membrane and descend into the cytoplasm. Therefore, we first examined the effect of actin inhibitors on the CME process. The analysis of the CCP lifetime in the presence of actin inhibitors revealed an inhibitory effect of the cortical actin network on the progress of CME. Cytochalasin B (an inhibitor of actin polymerization) and CK666 (an inhibitor of the Arp2/3 complex, which binds to F-actin and generates a branching point) both shortened the CCP lifetime, whereas jasplakinolide—which inhibits actin depolymerization and stabilizes the cortical actin network [36]—prolonged the lifetime (Fig 6A, S2 Table, S5–S7 Movies). In the presence of cytochalasin B, both the growing and stable phases shortened from 45 ± 34 s to 7 ± 12 s and from 88 ± 29 s to 59 ± 33 s, respectively, whereas there was little effect on the duration of the closing step (31 ± 9 to 22 ± 7 s) (Fig 6B, S3 Table, S8 Movie). This indicates that the collapse of the actin network accelerates CCP assembly and maturation, whereas the stabilization of the network inhibits the process. Dissecting the two-step closing motion also revealed that cytochalasin B and CK666 shortened the duration of the large aperture, whereas jasplakinolide prolonged it (Fig 6A), implying that actin dynamics accelerate the assembly of CCP-related proteins.

**Fig 7:**
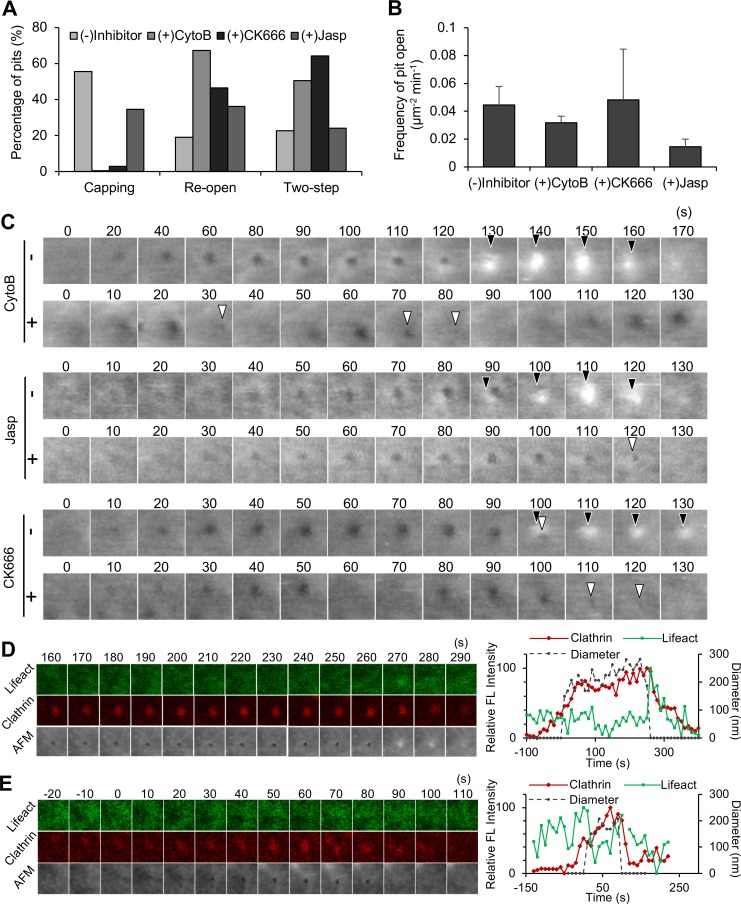
Role of actin in the closing step of CME. (A) The frequencies of capping, re-opening, and two-step motions were counted as described in Fig 5D in the absence or presence of cytochalasin B, CK666, or jasplakinolide. (B) The frequency of CCP formation was quantified in the absence or presence of the inhibitors and summarized. Error bars represent SD. (C) Time-lapse AFM images obtained in a living COS-7 cell before and after treatment with cytoB, CK666, or jasp. Image size: 0.5 × 0.5 μm^2^. Capping and the small aperture are indicated by black and white arrowheads, respectively. (D, E) A summary of actin assembly at a CCP from time-lapse hybrid imaging of COS-7 cells expressing EGFP-Lifeact and mCherry-CLCa. Fluorescence images for Lifeact and clathrin, as well as AFM images, are shown every 10 s (left panels). Image size: 1.2 × 1.2 μm^2^. Two representative CCPs are shown here. The CCP closes with (panel D) and without (panel E) capping. The signal intensities of the fluorescence spots (for Lifeact [green] and clathrin [red]) relative to their maximum values and the diameter of the membrane invagination in the AFM image (black) are plotted against time. Underlying data may be found in S1 Data. AFM, atomic force microscopy; CCP, clathrin-coated pit; CME, clathrin-mediated endocytosis; COS-7, CV-1 in origin with SV40 gene line 7; cytoB, cytochalasin B; EGFP, enhanced green fluorescent protein; EGFP-CLCa, EGFP-fused clathrin light chain a; FL, fluorescence; jasp, jasplakinolide.

In addition to the lifetime of the CCP, actin dynamics are involved in the closing motion of the CCP. The most striking effect of the inhibitors was a reduction of the capping motion and an increase in re-opening motions; cytochalasin B and CK666 drastically reduced the frequency of capping motions (from 56% to 0.4% by cytochalasin B and from 56% to 3% by CK666) and increased the frequency of re-opening motions (from 20% to 67% by cytochalasin B and from 20% to 47% by CK666) (Fig 7A and 7C, S4 Table) (see S9 Fig for other examples). The involvement of actin polymerization in membrane swelling was also demonstrated by ion-conductance microscopy [31] (see Discussion section for details). Jasplakinolide also showed a similar effect but to a smaller extent (Fig 7A and 7C). These observations are in good agreement with the result that the capping and re-opening motions are inversely related (Fig 5E) and that actin polymerization plays a role in an efficient and irreversible closing of the vesicle. In addition to the re-opening motions, two-step motions were also increased by cytochalasin B and CK666 treatments (Figs 7A and 6A), suggesting that two-step motions and actin polymerization are tightly coupled. Blocking actin depolymerization, but not polymerization, affected the frequency of CCP formation as previously reported [35], implying that the cortical actin layer also has an inhibitory effect on CCP formation.

To confirm that membrane swelling in the capping motion was induced by actin, we followed the localization of actin during CME. COS-7 cells simultaneously expressing Lifeact-GFP and mCherry-CLCa were subjected to time-lapse hybrid imaging. There were several variations in the actin signal depending on the basal level of actin around the CCP (Fig 7D and 7E, S9 Movie) (see S10 Fig for other examples). When the basal level was low, a burst of actin assembly was observed when the CCP closed. More precisely, it started to increase before the pit closed (−59 ± 18 s [mean ± SD]; *n* = 14), and peaked slightly after (2.5 ± 7 s [mean ± SD]; *n* = 15) the pit closure (Figs 3E and 7D). The burst of actin signal intensity was tightly correlated with the membrane swelling in the capping motion; the actin signal peaked when the membrane swelled. This is in good agreement with the result obtained with cytochalasin B (Fig 7A), in which addition of cytochalasin B reduced the frequency of the capping motion. On the other hand, when the basal actin level around the CCP was high, the signal first decreased during the growing and stable phases, then increased again toward the end of the CME (Fig 7E). In this case, membrane swelling was not observed. Taken together, these results demonstrate that actin depolymerization occurs in the growth and maturation phases of CME, and active actin assembly is required for the irreversible scission of the vesicle from the plasma membrane. Furthermore, the swelling of the membrane sometimes developed into a ruffle-like protrusion, even after the pit closure (S10 Fig), demonstrating that active polymerization of actin occurs around the CCP and generates local forces on the membrane.

### Dynamin is involved in the complete closure of CCPs

**Fig 8:**
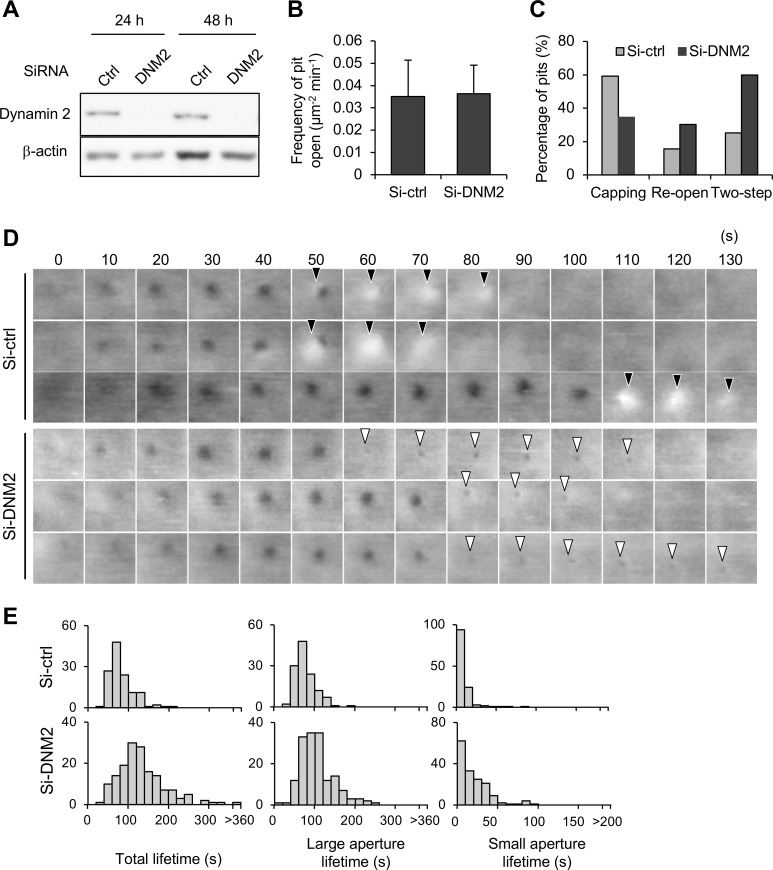
Effects of dynamin knockdown on CME. (A) Knockdown efficiency of dynamin 2 in COS-7 cells was examined by western blotting. Total cell lysates of COS-7 cells transfected with siRNA targeting the dynamin 2 gene (si-*DNM2*) or control siRNA (Luciferase, si-ctrl) were prepared 24 and 48 h after the transfection and were subjected to western blotting using anti-dynamin 2 and β-actin antibodies. The density of the band corresponding to dynamin was quantified using β-actin as a loading control. The knockdown efficiency was 88% after 24 h and 92% after 48 h. (B) The frequency of CCP formation was counted and compared in si-ctrl and si-*DNM2* cells. Error bars represent SD. (C) The frequency of capping, re-opening and two-step motions were analyzed in dynamin- and control-depleted cells as described in Fig 5D. (D) Time-lapse AFM images obtained in a living COS-7 cell transfected with si-ctrl or si-*DNM2*. Image size: 0.5 × 0.5 μm^2^. Capping and small apertures are indicated by black and white arrowheads, respectively. (E) The total lifetime and the durations of large and small apertures, measured in control- and dynamin-depleted cells, are summarized. Underlying data may be found in S1 Data. AFM, atomic force microscopy; CCP, clathrin-coated pit; CME, clathrin-mediated endocytosis; COS-7, CV-1 in origin with SV40 gene line 7; siRNA, small interfering RNA.

The involvement of other CCP-related proteins in morphological changes of the membrane was further investigated using RNA interference. Knockdown of dynamin 2 (Fig 8A)—in which 88% and 92% of dynamin 2 expression was suppressed after 24 and 48 h of transfection, respectively—did not affect the frequency of pit formation (Fig 8B) but reduced the occurrence of the capping motion from 59% to 35% (Fig 8C and 8D, S5 Table, S10 Movie). The effect was similar to what was observed in the presence of cytochalasin B (Fig 7A). However, in contrast to cytochalasin B and CK666, which increased both re-open and two-step motions, dynamin knockdown markedly increased the two-step motion but only slightly increased the re-open motion (Fig 8C). The detailed analysis of the two-step motion revealed a prolonged duration of the small aperture (Fig 8E, S3 Table). These results suggest that dynamin is involved in the capping formation as well as the complete closing of the CCP.

In addition to the prolonged duration of the small aperture (two-step motion), the duration of the large aperture was also slightly prolonged in the dynamin-knockdown cells, which resulted in a significant increase of the CCP population with total lifetime longer than 100 s (13% in control knockdown and 54% in dynamin knockdown) (Fig 8E, S6 Table). This is in good agreement with our observation that dynamin started to appear at the CCP during the growing phase (Fig 3C and 3D). These results suggested that dynamin is involved not only in the closing step but also in the assembly and maturation phases of the CCP, as was suggested in a previous study [25]. It should be noted that dynamin knockdown did not completely block the progress of CME, implying that a small amount of dynamin 2 slowly catalyzes the closing reaction or dynamin 1 is induced to compensate for the reduction of dynamin 2, as was demonstrated in the previous study [37,38] (see Discussion section for details).

## Discussion

Imaging the shape of the plasma membrane together with localization of proteins has been technically challenging. Although AFM was first applied to a living cell in 1992 [39] and a hybrid system of AFM and fluorescence microscope was available [40,41], their low scanning rate did not allow visualization of the endocytic process. Many other microscopic techniques such as high-frequency microrheology [42] and high-speed ion-conductance microscopy (HS-ICM) [31] have also been utilized for cell surface imaging and characterization. The recent development of HS-AFM with a sample-scanning system overcame the problem of limited time resolution and visualized the dynamics of cell surface structures with subsecond time resolution [43]. However, AFM with a sample-scanning configuration is not suitable for combining with high-resolution live-cell imaging of CLSM because this configuration moves the specimen (living cells) while the position of the cantilever is fixed. In this study, we utilized a tip-scanning type of HS-AFM coupled with CLSM and were able to successfully visualize morphological changes of the plasma membrane during CME in a living cell. Our AFM images revealed unique membrane structures at the end stage of CME as well as the role of CME-related proteins, especially actin and dynamin, in such morphological dynamics. Notably, some of these structures are similar to what were previously observed by different approaches (TIRF, ion conductance, etc.), demonstrating the fidelity of our imaging technique.

Recent advances in fluorescence microscopy enabled highly precise spatiotemporal analyses of proteins involved in CME in yeast [44,45] and animal cells [4]. They revealed the timings of protein assembly at the CCP and disassembly after the pit closure. On the other hand, the morphological changes of the plasma membrane during CME were mainly drawn based on EM snapshots of fixed and stained cells. The localization of a specific protein on the CCP has also been revealed by immune EM and by fluorescence EM. For instance, clathrin exists at the place where the membrane is slightly bent [11,12]. However, the time resolution of the snapshot analysis is limited and is not suitable for following membrane dynamics. In time-lapse fluorescence imaging, a complete pit closure was detected by a pH-sensitive fluorescent dye combined with fast exchange of the external medium between high- and low-pH solutions [4,46,47], but other morphological changes of the membrane—such as invagination and protrusion—could not be detected. Our time-lapse hybrid imaging of HS-AFM and CLSM rendered both the membrane morphology and protein localization with a time resolution of several seconds, which was particularly suitable for tracking the morphological changes of the CCP together with the assembly of specific proteins.

Our observations of fixed cells, unroofed cells, and living cells all revealed that the size of the CCPs varied between 150 and 400 nm, which is slightly larger than the CCPs in other cell lines. This is somehow similar to clathrin plaque, which is larger than CCPs and undergoes a different mechanism of endocytosis (50–500 nm in HeLa cells and 50–300 nm in skin melanoma cell line 2 [SK-MEL-2]) [12]. However, we believe that what we observed in this study were CCPs and not clathrin plaques because of the following two reasons. First, although the clathrin plaque is involved in endocytosis, it initially forms a flat sheet of clathrin on the flat plasma membrane without significant membrane invaginations. On the other hand, in our observations, the membrane started to invaginate approximately 30 s after clathrin started to assemble (Figs 2 and 3E). Second, a previous study reported that COS-7 cells contain clathrin plaques at the basal plasma membrane but not at the apical (nonadhering) surface [33].

Our image analysis of CME revealed several unique membrane dynamics at the end of the process, which is coupled with the function of related proteins (Fig 5): capping, a two-step motion, and re-opening. Capping occurred in most of the CME events (approximately 60%) and was mediated by actin (Fig 7) and dynamin (Fig 8). A region of the adjacent membrane swelled and covered over the CCP (Figs 5 and 7). The peak actin signal (GFP-Lifeact) corresponded with the timing of membrane swelling (Fig 7D), and the inhibition of actin polymerization by inhibitors (cytochalasin B and CK666) perturbed the capping motion (Fig 7A), indicating that the membrane swelling is caused by rapid and local actin polymerization. These characteristics of capping seem to be similar to membrane protrusions observed by HS-ICM combined with CLSM [31]; small membrane protrusions (caps) were frequently (101 out of 145 at 28 °C) observed beside the pit at the end of CME. The cap was abolished when actin polymerization was inhibited by latrunculin B [31]. Because such membrane protrusion was reported in several other studies [31,43], this could be a general mechanism of CME. In addition to this observation, we found that this capping motion was related to other closing motion (re-open) (Fig 7) and also to the function of dynamin (Fig 8), suggesting functional interaction between actin and dynamin at the closing step of CME (see later section for further discussion on dynamin). It is noted that most of the membrane swelling occurs at one side of the CCP and moves across the pit towards the opposite side (Figs 5 and 7), which is reminiscent of actin comet tails formed behind the motile *Listeria monocytogenes* [48,49]. We could not find any preference in the direction of the capping. It might be the case that a sudden burst of actin polymerization at a certain point on the CCP induces membrane swelling. These results clearly support the idea that a short burst of actin polymerization produces membrane swelling beside the pit.

In addition to capping, re-open motion was frequently observed in our AFM images. There are 3 possible interpretations of this events: (i) a new CCP was formed at the same position of the membrane after the previous vesicle was budded off (re-formation), (ii) the CCP is still connected to the plasma membrane with an unresolvable small aperture and reversibly changes the aperture size (re-widening), or (iii) the CCP is completely separated from the plasma membrane and then reversibly fuses back to the original position (re-fusion). In the first case (re-formation), the motion has several similarities to what was previously reported as the “hot spot,” which is approximately 400 nm in diameter and sequentially produces CCPs [34] with multiple cycles of dynamin polymerization [32]. It can be speculated that clathrin and other proteins remained in the hot spot and efficiently recruited proteins necessary for another round of CME. In our observation, clathrin also remained after the closure of the first CCP and started to increase again during the second growing phase (Fig 5C). The rate of the clathrin assembly was very similar between first and second CCP assembly (Fig 5C). These results strongly suggest that the re-open motion we observed in AFM could be re-formation of the CCP at the same spot soon after the preceding pit is closed. However, this could not totally exclude the possibility of re-fusion and re-widening models. Further analyses with higher spatiotemporal resolutions of both AFM and fluorescence signal will provide a clearer answer to this question.

It is noted that inhibition of actin dynamics not only decreased the capping motion but also increased the occurrence of re-opening motions (Fig 7A). There could be several possible explanations for this observation. If the re-open motion is a fusion of the vesicle back to the plasma membrane (re-fusion) as we discussed before, this result suggests that actin dynamics are required for irreversible detachment of the CCV from the plasma membrane. In one possible scenario, actin polymerizes around the vesicle and provides a driving force to push the vesicle into the cytoplasm by interacting with myosin in the cell cortex. There are a number of reports of nonmuscle myosins in the cell cortex [50–52], which are involved in various molecular events at the cell surface. Another scenario is that actin assembles near the plasma membrane but does not attach to the vesicle. Newly assembled actin filaments between the plasma membrane and the vesicle may spatially hinder the reversible fusion of the vesicle to the plasma membrane, which consequently pushes the vesicle into the cytoplasm, ultimately leading to endosome fusion, as was suggested by previous studies [53,54]. If the re-open motion is re-formation of a new vesicle (hot spot), this result suggests that actin dynamics have a negative effect on the formation of a new vesicle. However, our result that the inhibition of actin did not affect the number of newly formed CCPs (Fig 7B) does not support this possibility. To clarify the mechanism of the re-open motion by actin inhibitors, further studies will be required.

In addition to the closing motion, we found a role of actin dynamics in the growing phase of the CCP. Our observation that destruction of the actin network by cytochalasin B or CK666 accelerated the CME process (shortened the lifetime of open apertures) and stabilization of the network by jasplakinolide prolonged the lifetime (Fig 6) suggests that the cortical actin layer has an inhibitory effect on the progress of CME. This could be explained by the following 3 mechanisms. The first possibility is that the assembly of CCP protein components at the plasma membrane is inhibited by the cortical actin layer. Because the cortical layer is supposed to be similar to a hydrogel state, the diffusion of cytoplasmic proteins through the cortex is also supposed to be slow. The second possibility is that the cortical actin layer spatially inhibits the growth of the CCP. As the size of the CCP grows, neighboring actin filaments must be excluded. Although it is not clear whether the exclusion is mediated by physical force or an enzymatic process [55,56], the progress of CME is tightly coupled with the dynamics of the cortical actin network. The third possibility is that the actin cortex generates membrane tension that inhibits the progress of CME. Disruption of actin cortex reduces the membrane tension and accelerates the pit formation by clathrin coating.

The role of actin dynamics in CME has been debated in previous studies [53,57]. The cortical actin layer is known to be actively involved not only in endocytosis but also exocytosis. In a secretion process of neuronal cells, the cortical actin network is involved in (i) tethering secretory vesicles [58–63], (ii) providing a platform for directed movement toward the plasma membrane [64], and (iii) facilitating the generation of new release sites [65–68]. In endocytosis, actin is known to localize to the CCP in both yeast and mammalian cells [44,45]. Although several models have been proposed for the function of actin in CME, many details remain obscure. Our results demonstrate not only the involvement of actin in capping formation but also functional interaction of actin and dynamin in the closing step of CME.

In addition to actin, we demonstrated the role of dynamin in the closing motion (Fig 8). Knock down of dynamin reduced the capping and re-opening motions and increased the two-step motion (Fig 8), which is partly similar to the effect of actin inhibition (Fig 7). This effect can be partially explained by a feedback mechanism between actin and dynamin recruitment, which was previously reported [5]; dynamin knockdown decreased the accumulation rate of actin, which resulted in the decrease of the capping motion. In addition, the loss of dynamin apparently prolonged the duration of a smaller aperture size in the two-step motion (Fig 8), suggesting that dynamin plays a role not in the initial narrowing of the aperture but in the complete scission of the vesicle. This is compatible with the known function of dynamin: it binds to the neck of the CCP and catalyzes membrane scission [69,70]. On the other hand, a recent in vitro study reported that actin and BAR domain proteins, but not dynamin, are essential for membrane scission [71]. Therefore, it might be the case that the initial narrowing of the pit aperture is mediated by actin and BAR domain proteins, and the final scission step might be accelerated by a catalytic function of dynamin. In such a case, the inhibition of individual proteins would not completely abolish the complete process but would only slow down the progress.

We found that knock down of dynamin also prolonged the assembly and/or maturation phases of CME (Fig 8). Indeed, dynamin appeared on the CCP just prior to the initiation of membrane invagination and kept accumulating as the pit grew (Fig 3) [72]. The role of dynamin in the assembly and maturation phases has been debated in previous studies [5,25]. Because the catalytic activity of dynamin is regulated by nucleotide-based mechanisms [70], the assembly of dynamin at the CCP may not, in itself, induce any membrane deformations. Considering the fact that the dynamin knockdown slowed down the progress of CME, it might be the case that dynamin is necessary for recruiting other protein machineries to the CCP. Further analyses are required for elucidating the role of dynamin in the whole process of CME. Another possibility might be an activation of dynamin 1, which was recently reported in non-neuronal cells [38, 39]; the dynamin 1—which is presumed to be a neuron-specific isoform of dynamin but was recently found to be expressed in many non-neuronal cell lines—was activated in non-neuronal cells when CME was dysregulated with the expression of a truncated mutant of adaptor protein. Therefore, at nonphysiological low temperature, dynamin 1 might be activated and compensate for the reduction of dynamin 2. Further investigation is required to reveal functional interaction between 2 isoforms of dynamin in CME.

All of the observations described in this study were conducted at 28 °C. As demonstrated in previous studies, endocytic activity is largely affected by temperature, especially in neuronal cells [73–75]. This is partly due to the reduction of membrane fluidity and of catalytic activity of proteins involved. Actin polymerization is reduced in cultured cells at nonphysiological temperature [76,77]. Because the actin polymerization promoted CME (inhibition of actin dynamic elongated the lifetime, Fig 6), imaging at nonphysiological temperature may elongate the lifetime of CCPs, as was discussed before [31]. Therefore, it is highly intriguing to observe dynamic morphologies of CCPs at physiological temperature and reveal the involvement of membrane and protein dynamics in the CME process. For this purpose, the establishment of a stable imaging system of HS-AFM at 37 °C, as well as a higher scanning rate, is necessary.

## Materials and methods

### Materials

COS-7 cells were purchased from DS Biopharma Medical (EC87021302-F0). Cytochalasin B was purchased from Sigma-Aldrich (St. Louis, MO), and jasplakinolide was purchased from Abcam (Cambridge, United Kingdom). The reagents were added to the culture medium at final concentrations of 2 μM for cytochalasin B and 1 μM for jasplakinolide. HEPES-NaOH (pH 7.0–7.6), which was used to maintain a constant pH of the medium during AFM observation, was purchased from Sigma-Aldrich. The mammalian expression vectors encoding Dyn2-pmCherryN1 and epsin 2-pmCherryC1 were gifts from Christien Merrifield (Addgene #27689 and #27673, respectively), and the vector for EGFP-Lifeact expression was a kind gift from Dr. Mineko Kengaku (Kyoto University, Kyoto, Japan). Silencer select siRNA targeting *DNM2* (#s4212) and siRNA targeting Luciferase (#12935–146) were purchased from Ambion (Waltham, MA) and ThermoFisher Scientific (Waltham, MA), respectively. Transfection reagents Lipofectamin2000 and Effectene were purchased from ThermoFisher Scientific and Qiagen (Hilden, Germany), respectively. Anti-dynamin 2 antibody was from Cell Signaling Technology (Danvers, MA), and PVDF membrane was from Bio-Rad Laboratories (Hercules, CA).

### Cell culture, transfection and fixation

At 1 or 2 d before AFM imaging, COS-7 monkey kidney–derived fibroblast-like cells were seeded on a poly-L-lysine–coated glass slide and grown at 37 °C with 5% CO_2_ in Dulbecco’s Modified Eagle Medium (DMEM) supplemented with 10% FBS. AFM imaging was performed in DMEM supplemented with 10% FBS and 10 mM HEPES-NaOH (pH 7.0–7.6). cDNAs encoding human CLCa (CLTA, NM_007096) and caveolin1 (CAV1, NM_00172895) were amplified by RT-PCR and cloned into the vector pEGFP-C3 (Clontech, Fremont, CA) to create fused proteins with EGFP. The plasmids were introduced into cells using Effectene Transfection Reagent according to the manufacturer’s protocol. At 24 to 48 h after transfection, the cells were used for AFM imaging. Expression of the fusion protein was confirmed by fluorescence signals from the cells. For experiments with fixed cells, the cells were fixed with 5% paraformaldehyde in PBS for 15 min at room temperature and washed with PBS. For unroofing cells on a cover glass, cells were mildly sonicated, as described in the previous study [22], and then fixed.

### RNA interference

Cells were transfected with siRNAs using Lipofectamine 2000 (Invitrogen, Carlsbad, CA) following the manufacturer’s instructions and were harvested after 24 to 48 h for analysis by SDS-PAGE and immunoblotting, using PVDF membranes. Each membrane was cut into 2 halves at the protein size of 60 kDa; the upper half was blotted with anti-dynamin 2 antibody, and the bottom half was detected with anti-β-actin antibody.

### AFM imaging and data analysis

BIXAM (Olympus Corporation, Tokyo, Japan)—which is a tip-scan–type HS-AFM unit combined with an inverted fluorescence/optical microscope (IX83; Olympus) equipped with a phase contrast system and a confocal unit (FV1200; Olympus)—was used for this study. The tip-scan HS-AFM imaging system was developed based on a previous study [20]. In brief, the modulation method was set to phase modulation mode to detect tip–sample interactions. An electron beam–deposited sharp cantilever tip with a spring constant of 0.1 N m^−1^ (USC-F0.8-k0.1, a customized cantilever from Nanoworld [Neuchâtel, Switzerland]) was used. All observations were performed at 28 °C. The AFM tip was aligned with confocal views as described in the Results section. The images from the confocal microscope and AFM were simultaneously acquired at a scanning rate of 10 s/frame. The captured sequential images were overlaid by using AviUTL (http://spring-fragrance.mints.ne.jp/aviutl/) based on the tip position. The fluorescence intensity was quantified by Image J software (http://rsbweb.nih.gov/ij/). The lifetime of the pit was analyzed with Metamorph imaging software (Molecular Devices, San Jose, CA). The diameter/height of the pit or membrane swelling region was obtained using AFM Scanning System Software Version 1.6.0.12 (Olympus).

For the measurement of the x-y offset between pit and clathrin fluorescence spots, the distance between the centroids of the AFM invagination and the fluorescence spot were measured in ImageJ. A diffusion coefficient of the pit was calculated based on the position of the pit. In the calculation, the effect of drift defined as a displacement of caveolin fluorescence spots in the fluorescence movie was corrected by subtracting a drift from pit position. For time-lapse analysis of AFM and fluorescence images of the CCPs, the time when the plasma membrane started to invaginate and when the pit completely closed on AFM images are defined as t = 0, and the fluorescence signal intensity was plotted against this time scale.

### Statistical analysis

Data presented as graphs are from 3 independent experiments. The number of total CCPs analyzed for each analysis is specified in figure legends or the main text. Statistical analysis was performed by two-way analysis of variance followed by Student *t* test.

## Supporting information

S1 Fig Aligning confocal and AFM images. (A) Scanning electron microscopy (SEM) images of a cantilever equipped with an EBD tip with tilt angle of 12°. Scale bar, 5 μm. Note that the cantilever is held on the AFM head unit with a tilt angle of 102° (from the x-y plane) so that the relative tip–sample angle (*θ*) is 90°. This setup makes it possible to precisely determine the position of the AFM tip. Scale bar, 2 μm. (B) Determining the position of the AFM probe in a fluorescence image. While the AFM probe was attached on the glass surface without scanning, the autofluorescence signal of the probe was imaged by the confocal scanning unit. The observed fluorescence spot (arrowhead in the middle panel) is defined as an origin of the fluorescence image plane (x = 0, y = 0) and used to define the optical axis (left panel). The position of a fluorescence spot derived from EGFP-CLCa was determined on this axis. On the other hand, the scanning area of the AFM scanner covers the area of (−3, 2.25) (left top), (3, 2.25) (right top), (3, −2.25) (right bottom), and (−3, −2.25) (left bottom) (all right panel). By aligning the axis from both images, the x, y position of the AFM image and that of the confocal fluorescence image could be merged. AFM, atomic force microscopy; EBD, electron beam–deposited; EGFP, enhanced green fluorescent protein; EGFP-CLCa, EGFP-fused clathrin light chain a.
(TIF)

S2 Fig Characterization of membrane morphologies in AFM images. (A, B) Diameter of the CCP in AFM images. The cross-section profile across the CCP (shown with dotted line in panel A) was produced using the AFM Scanning System Software Version 1.6.0.12 (Olympus) and is shown in panel B. The diameter of the pit was defined as the distance in horizontal direction between 2 points at the edge of the invagination (shown with red circles). The geometry of the CCP observed by AFM (150–400 nm in diameter) was slightly larger than what was previously observed by EM^12^ (20–175 nm in inner diameter). In the EM analysis, a fixed cell sample was sliced, and measurements of the inner diameter (neck width) were obtained from these sections. The apparent difference in the values is most likely due to the constraints of the AFM image analysis because only the outer diameter could be measured in AFM; with our technique, the inner diameter of the neck could not be imaged and measured. Ion-conductance microscopy also identified the CCP on a living cell surface, with a size range of 50–300 nm^61^, which roughly matches the results for AFM and EM. (C, D) Measurement of membrane height in AFM images. The cross-section profile across the CCP (shown with a dotted line in panel C) was produced by the same procedure described in panels A and B and shown in panel D. The height of the membrane swelling was defined as a distance in the z-direction between the highest point (gray circle) and the average of 2 basal levels (red circles). Underlying data may be found in S1 Data. AFM, atomic force microscopy; CCP, clathrin-coated pit; EM, electron microscopy.
(TIF)

S3 Fig Offset of the CCP and clathrin fluorescence spot during time-lapse hybrid imaging. (A) Sequential AFM images (top) and fluorescence images of EGFP-CLCa (middle) were overlaid by the procedure described in S1 Fig (bottom). The AFM images were trimmed from the 6.0 × 4.5 μm^2^ image. Image size is 600 × 600 nm^2^. (B) The center positions of the clathrin spot in the fluorescence image (green) and the CCP spot in the AFM image (black) were plotted for 100 s on the merged image. (C) The offset (x, y, and xy) between the clathrin spot and the CCP spot was measured in each frame and plotted against time. Underlying data may be found in S1 Data. AFM, atomic force microscopy; CCP, clathrin-coated pit; EGFP, enhanced green fluorescent protein; EGFP-CLCa, EGFP-fused clathrin light chain a.
(TIF)

S4 Fig Effects of tip-interaction during AFM imaging. (A) Effect of a scanning parameter on the morphology of CCP. The time-lapse imaging of the CCP was conducted by changing the driving amplitude. As shown here, increasing the amplitude by 5% resulted in the image with clearer cytoskeletal pattern. In this case, the diameter of the CCP (arrow) changed by 9%. (B) An example of tip skipping. During the observation of the plasma membrane, tip skipping, which is indicated by an arrow in the left panel, occurs. The section profile (right panel) across the skipping region (dotted line in the left panel) indicates that it occurs at 1 or 2 consecutive lines and therefore does not affect morphological analyses of the CCP. Underlying data may be found in S1 Data. AFM, atomic force microscopy; CCP, clathrin-coated pit.
(TIF)

S5 Fig Procedures for characterizing CCPs. (A) Defining growing, stable, and closing phases. The fluorescence signal intensity of clathrin relative to the maximum value (grey) was first subjected to smoothing by 3 frame windows (green). The stable phase was defined when the slope of the averaged profile (red) was within −0.05 and +0.1 (/frame) for more than 4 frames. The growing and closing phases were defined as the period before and after the stable phase. (B) Analysis of closing step with a higher scanning rate at 1 s/frame (C) Time-lapse plot of the area of the pit. The pit that was analyzed in Fig 2B was re-analyzed to measure the pit area. The time-lapse AFM images were sequentially processed with Median Filter, Top Hat (Circle, Diameter: 20 pixel) and Open-Close filter (Circle, Diameter: 6 pixel) by MetaMorph software. Underlying data may be found in S1 Data. AFM, atomic force microscopy; CCP, clathrin-coated pit.
(TIF)

S6 Fig Additional examples of hybrid time-lapse imaging of CCPs (EGFP-CLCa and mCherry-epsin). (A, B) Additional examples of hybrid time-lapse imaging of CCPs in a living COS-7 cell transiently co-expressing EGFP-CLCa and mCherry-epsin. Image size: 1.2 × 1.2 μm^2^. CCP, clathrin-coated pit; COS-7, CV-1 in origin with SV40 gene line 7; EGFP, enhanced green fluorescent protein; EGFP-CLCa, EGFP-fused clathrin light chain a.
(TIF)

S7 Fig Additional examples of hybrid time-lapse imaging of a CCP (EGFP-CLCa and Dyn2-mCherry). (A, B) Additional examples of hybrid time-lapse imaging of CCPs in a living COS-7 cell transiently co-expressing EGFP-CLCa and Dyn2-mCherry. Image size: 1.2 × 1.2 μm^2^. CCP, clathrin-coated pit; COS-7, CV-1 in origin with SV40 gene line 7; EGFP, enhanced green fluorescent protein; EGFP-CLCa, EGFP-fused clathrin light chain a.
(TIF)

S8 Fig Coexistence of two-step and capping in closure of CCPs. Sequential AFM images taken at 2-s intervals. Membrane swelling and small aperture are indicated by black and white arrowheads, respectively. Image size: 1.2 × 1.2 μm^2^. CCP, clathrin-coated pit.
(TIF)

S9 Fig Effect of actin inhibitors (cytoB, jasp, and CK666) on the closing motion of CCP. Time-lapse AFM images obtained in a living COS-7 cell before and after treatment with cytochalasin B, jasplakinolide, or CK666. Image size: 0.5 × 0.5 μm^2^. Capping and small apertures are indicated by black and white arrowheads, respectively. AFM, atomic force microscopy; CCP, clathrin-coated pit; COS-7, CV-1 in origin with SV40 gene line 7; cytoB, cytochalasin B; jasp, jasplakinolide.
(TIF)

S10 Fig Additional examples of hybrid time-lapse imaging of CCPs (Lifeact-GFP and mCherry-CLCa). (A, B, C) Additional examples of hybrid time-lapse imaging of CCPs in a living COS-7 cell transiently co-expressing Lifeact-GFP and mCherry-CLCa. Image sizes in panels A and B: 1.2 × 1.2 μm^2^. Image size in panel C: 1.0 × 2.3 μm^2^. Image size in panel D: 1.74 × 1.23 μm^2^. In (C) and (D), a membrane swelling developed into a large ruffle-like protrusion. The actin signal was colocalized with such ruffle-like protrusions. CCP, clathrin-coated pit; CLCa, clathrin light chain a; COS-7, CV-1 in origin with SV40 gene line 7; GFP, green fluorescent protein.
(TIF)

S1 Table Percentage of closing motion of CCPs in cells expressing EGFP-fused CLCa. The values are presented as mean ± SD.
(XLSX)

S2 Table Lifetime of CCPs in inhibitor-treated cells and the number of cells and CCPs used to obtain the values. The values are presented as mean ± SD.
(XLSX)

S3 Table Length of each phase in cytochalasin B–treated cells and the number of CCPs and cells used to obtain the values for each data set. The values are presented as mean ± SD.
(XLSX)

S4 Table Percentage of closing motion of CCPs in inhibitor-treated cells and CCPs used to obtain the values. The values are presented as mean ± SD.
(XLSX)

S5 Table Percentage of closing motion of CCPs in siRNA-transfected cells and the number of cells and CCPs used to obtain the values. The values are presented as mean ± SD.
(XLSX)

S6 Table Lifetime of CCPs in siRNA-transfected cells and the number of cells and CCPs used to obtain the values. The values are presented as mean ± SD.
(XLSX)

S1 Movie Hybrid time-lapse imaging of COS-7 cells expressing EGFP-CLCa. Images were obtained every 10 s and aligned by the procedure described in S1 Fig. Image size: 6.0 × 4.5 μm^2^.
(MOV)

S2 Movie Hybrid time-lapse imaging of COS-7 cells expressing EGFP-CLCa and mCherry-epsin. Images were obtained every 10 s and aligned by the procedure described in S1 Fig. Image size: 6.0 × 4.5 μm^2^.
(MOV)

S3 Movie Hybrid time-lapse imaging of COS-7 cells expressing EGFP-CLCa and Dyn2-m-Cherry. Images were obtained every 10 s and aligned by the procedure described in S1 Fig. Image size: 6.0 × 4.5 μm^2^.
(MOV)

S4 Movie Hybrid time-lapse imaging of COS-7 cells expressing EGFP-CLCa and mCherry-caveolin1. Images were obtained every 10 s and aligned by the procedure described in S1 Fig. Image size: 6.0 × 4.5 μm^2^.
(MOV)

S5 Movie Time-lapse imaging of COS-7 cells before and after treatment with cytochalasin B. Images were obtained every 10 s. Image size: 6.0 × 4.5 μm^2^.
(MOV)

S6 Movie Time-lapse imaging of COS-7 cells before and after the treatment with CK666. Images were obtained every 10 s. Image size: 6.0 × 4.5 μm^2^.
(MOV)

S7 Movie Time-lapse imaging of COS-7 cells before and after the treatment with jasplakinolide. Images were obtained every 10 s. Image size: 6.0 × 4.5 μm^2^.
(MOV)

S8 Movie Hybrid time-lapse imaging of COS-7 cells expressing EGFP-CLCa after the treatment with cytochalasin B. Images were obtained every 10 s and aligned by the procedure described in S1 Fig. Image size: 6.0 × 4.5 μm^2^.
(MOV)

S9 Movie Hybrid time-lapse imaging of COS-7 cells expressing mCherry-CLCa and LifeAct-GFP. Images were obtained every 10 s and aligned by the procedure described in S1 Fig. Image size: 6.0 × 4.5 μm^2^.
(MOV)

S10 Movie Time-lapse imaging of COS-7 cells transfected with either si-ctrl RNA or si-*DNM2* RNA. Images were obtained every 10 s. Image size: 6.0 × 4.5 μm^2^.
(MOV)

S1 Data Raw data for Fig 2B and 2C; Fig 2B, 2D and 2E; Fig 4B and 4D; Fig 5A–5F; Fig 6A and 6B; Fig 7A, 7B, 7D and 7E; Fig 8B, 8C and 8E; S2B and S2D Fig; S3C Fig; S4B Fig; S5A–S5C Fig. (XLSX)

## Abbreviations

AFM: atomic force microscopy
a.u.: arbitrary unit
BAR: Bin-amphiphysin-Rvs17
CCP: clathrin-coated pit
CCV: clathrin-coated vesicle
CLSM: confocal laser scanning microscopy
CME: clathrin-mediated endocytosis
COS-7: CV-1 in origin with SV40 gene line 7
DMEM: Dulbecco’s Modified Eagle Medium
EGFP: enhanced green fluorescent protein
EGFP-CLCa: EGFP-fused clathrin light chain a
EM: electron microscopy
FBS: fetal bovine serum
GAK: G-associated kinase
HS-AFM: high-speed AFM
HS-ICM: high-speed ion-conductance microscopy
MSD: mean square displacement
n.d.: not determined
siRNA: small interfering RNA
SK-MEL-2: skin melanoma cell line 2
