# Supplementary figures and images for: Morphological changes of plasma membrane and protein assembly during clathrin-mediated endocytosis
Source: PLoS Biol. 2018 May 3;16(5):e2004786. doi: 10.1371/journal.pbio.2004786 (PMC5953504; doi:10.1371/journal.pbio.2004786)

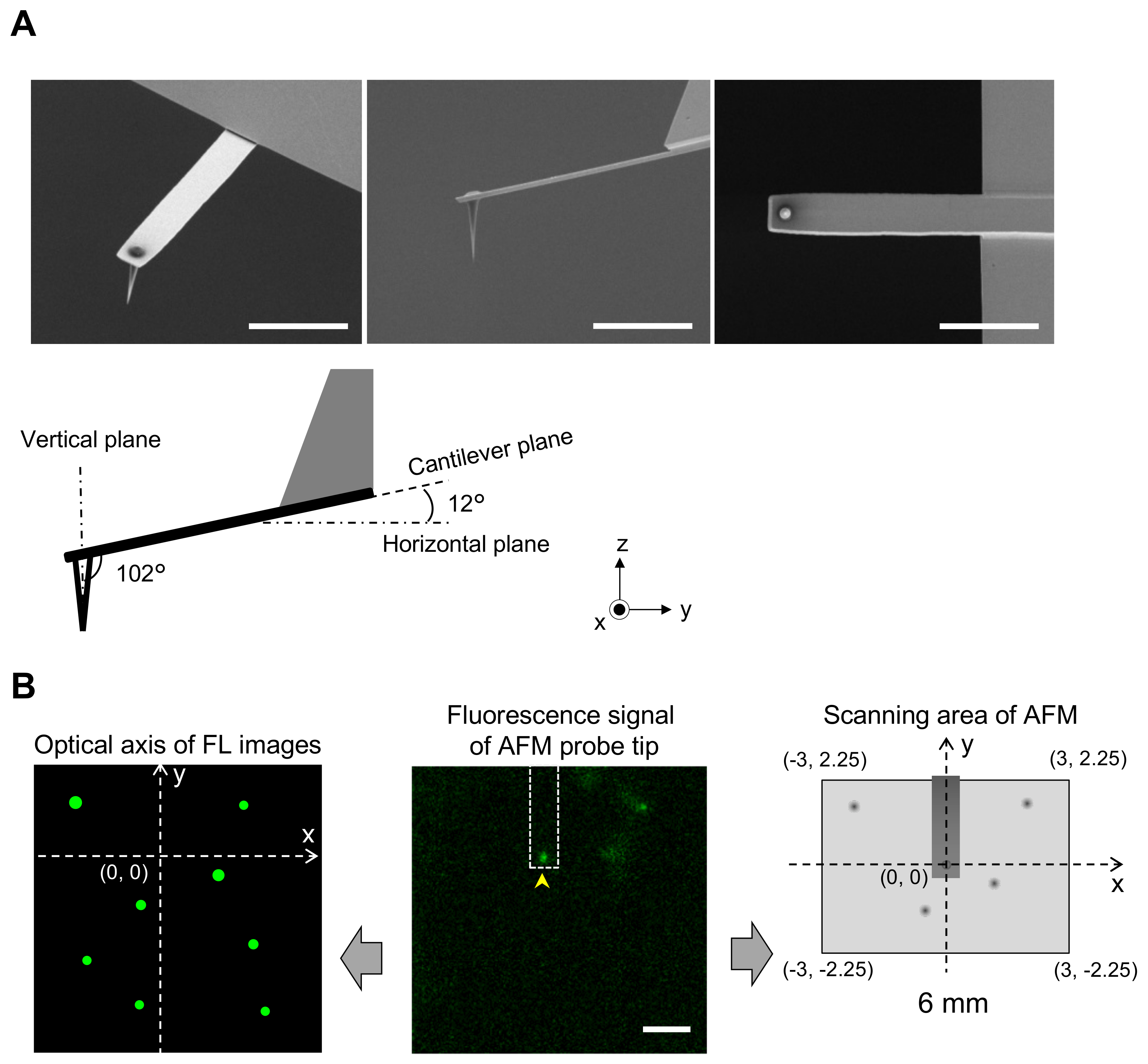

Supplement: S1 Fig — (A) Scanning electron microscopy (SEM) images of a cantilever equipped with an EBD tip with tilt angle of 12°. Scale bar, 5 μm. Note that the cantilever is held on the AFM head unit with a tilt angle of 102° (from the x-y plane) so that the relative tip–sample angle (θ) is 90°. This setup makes it possible to precisely determine the position of the AFM tip. Scale bar, 2 μm. (B) Determining the position of the AFM probe in a fluorescence image. While the AFM probe was attached on the glass surface without scanning, the autofluorescence signal of the probe was imaged by the confocal scanning unit. The observed fluorescence spot (arrowhead in the middle panel) is defined as an origin of the fluorescence image plane (x = 0, y = 0) and used to define the optical axis (left panel). The position of a fluorescence spot derived from EGFP-CLCa was determined on this axis. On the other hand, the scanning area of the AFM scanner covers the area of (−3, 2.25) (left top), (3, 2.25) (right top), (3, −2.25) (right bottom), and (−3, −2.25) (left bottom) (all right panel). By aligning the axis from both images, the x, y position of the AFM image and that of the confocal fluorescence image could be merged. AFM, atomic force microscopy; EBD, electron beam–deposited; EGFP, enhanced green fluorescent protein; EGFP-CLCa, EGFP-fused clathrin light chain a. (TIF) [file pbio.2004786.s001.tif]

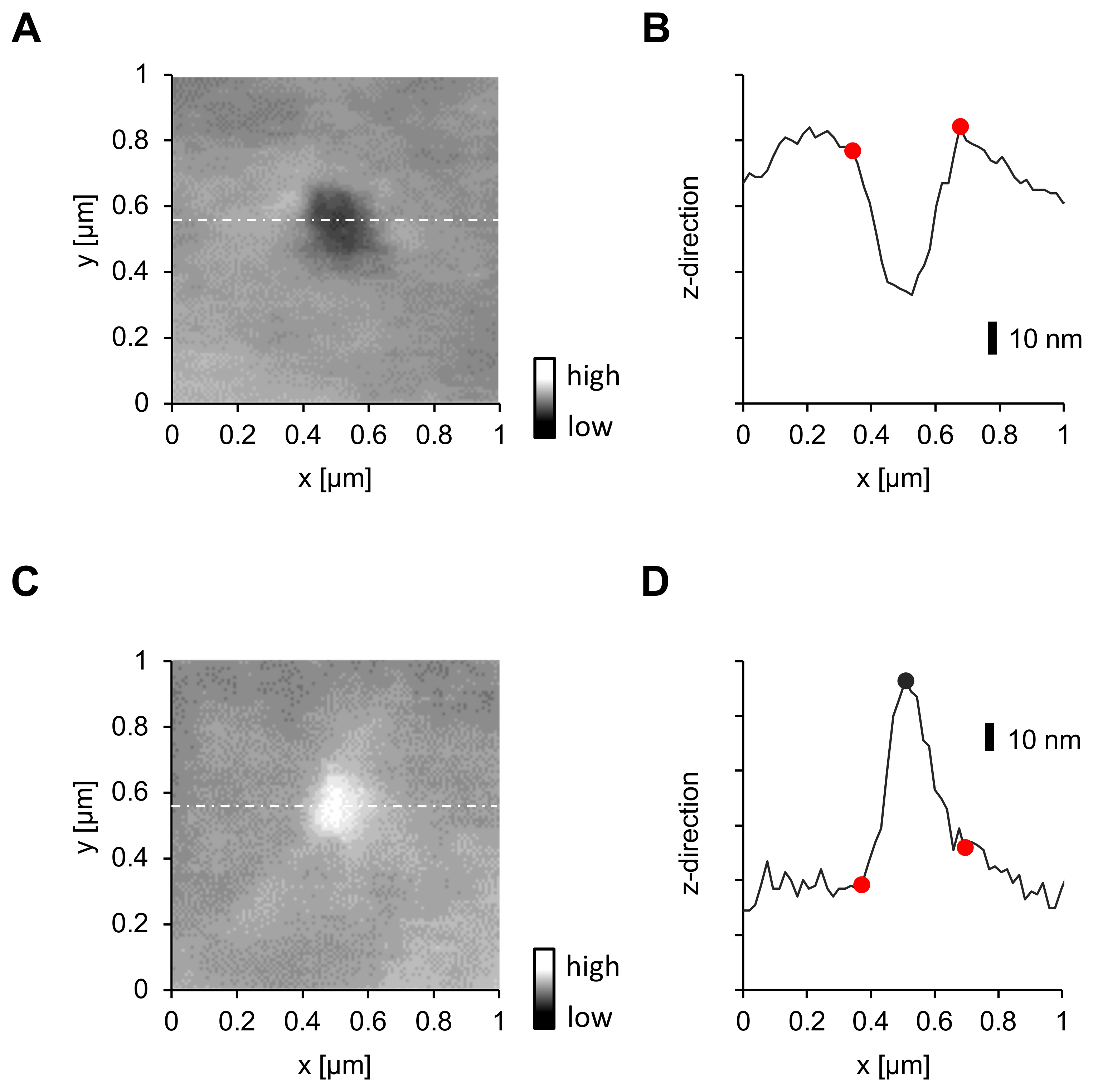

Supplement: S2 Fig — (A, B) Diameter of the CCP in AFM images. The cross-section profile across the CCP (shown with dotted line in panel A) was produced using the AFM Scanning System Software Version 1.6.0.12 (Olympus) and is shown in panel B. The diameter of the pit was defined as the distance in horizontal direction between 2 points at the edge of the invagination (shown with red circles). The geometry of the CCP observed by AFM (150–400 nm in diameter) was slightly larger than what was previously observed by EM12 (20–175 nm in inner diameter). In the EM analysis, a fixed cell sample was sliced, and measurements of the inner diameter (neck width) were obtained from these sections. The apparent difference in the values is most likely due to the constraints of the AFM image analysis because only the outer diameter could be measured in AFM; with our technique, the inner diameter of the neck could not be imaged and measured. Ion-conductance microscopy also identified the CCP on a living cell surface, with a size range of 50–300 nm61, which roughly matches the results for AFM and EM. (C, D) Measurement of membrane height in AFM images. The cross-section profile across the CCP (shown with a dotted line in panel C) was produced by the same procedure described in panels A and B and shown in panel D. The height of the membrane swelling was defined as a distance in the z-direction between the highest point (gray circle) and the average of 2 basal levels (red circles). Underlying data may be found in S1 Data. AFM, atomic force microscopy; CCP, clathrin-coated pit; EM, electron microscopy. (TIF) [file pbio.2004786.s002.tif]

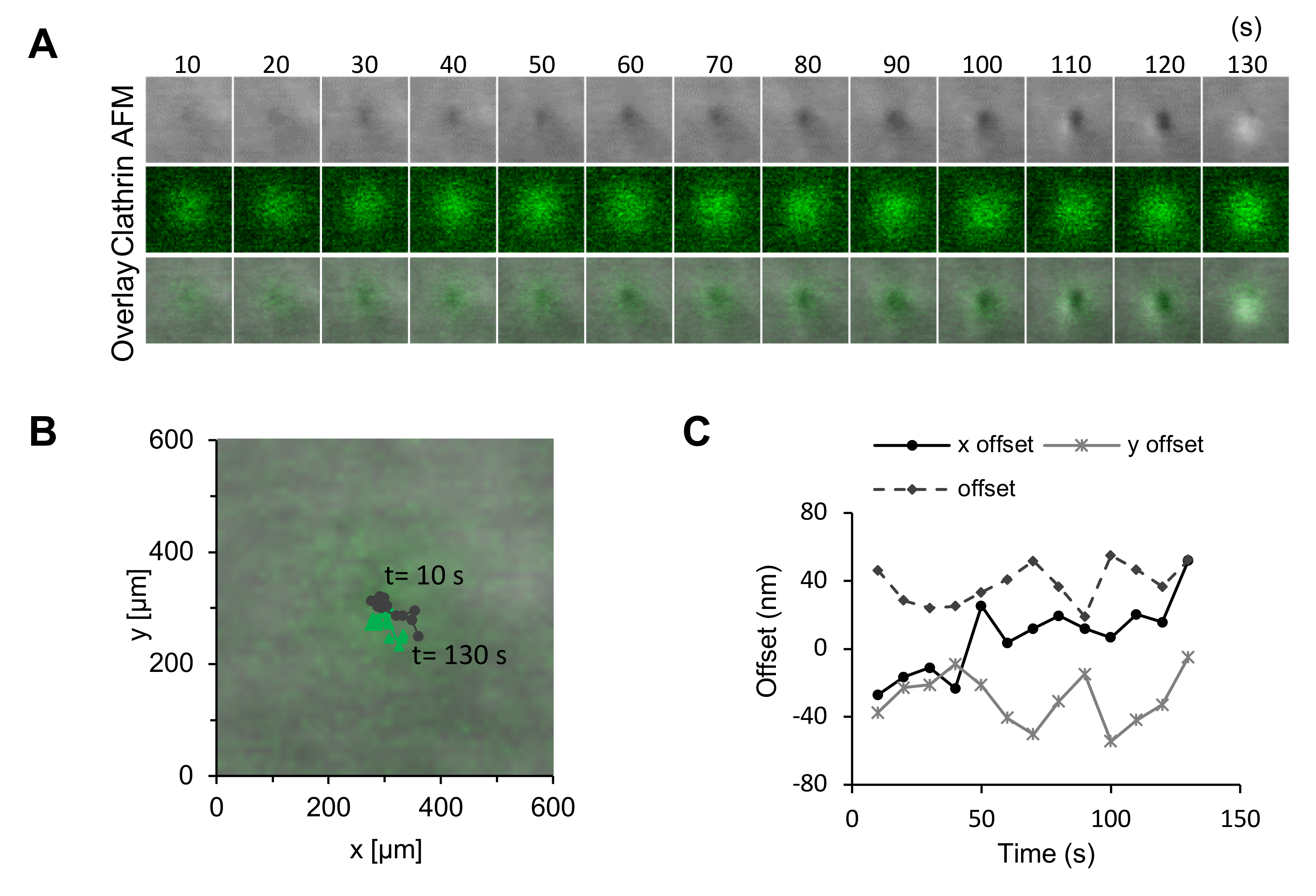

Supplement: S3 Fig — (A) Sequential AFM images (top) and fluorescence images of EGFP-CLCa (middle) were overlaid by the procedure described in S1 Fig (bottom). The AFM images were trimmed from the 6.0 × 4.5 μm2 image. Image size is 600 × 600 nm2. (B) The center positions of the clathrin spot in the fluorescence image (green) and the CCP spot in the AFM image (black) were plotted for 100 s on the merged image. (C) The offset (x, y, and xy) between the clathrin spot and the CCP spot was measured in each frame and plotted against time. Underlying data may be found in S1 Data. AFM, atomic force microscopy; CCP, clathrin-coated pit; EGFP, enhanced green fluorescent protein; EGFP-CLCa, EGFP-fused clathrin light chain a. (TIF) [file pbio.2004786.s003.tif]

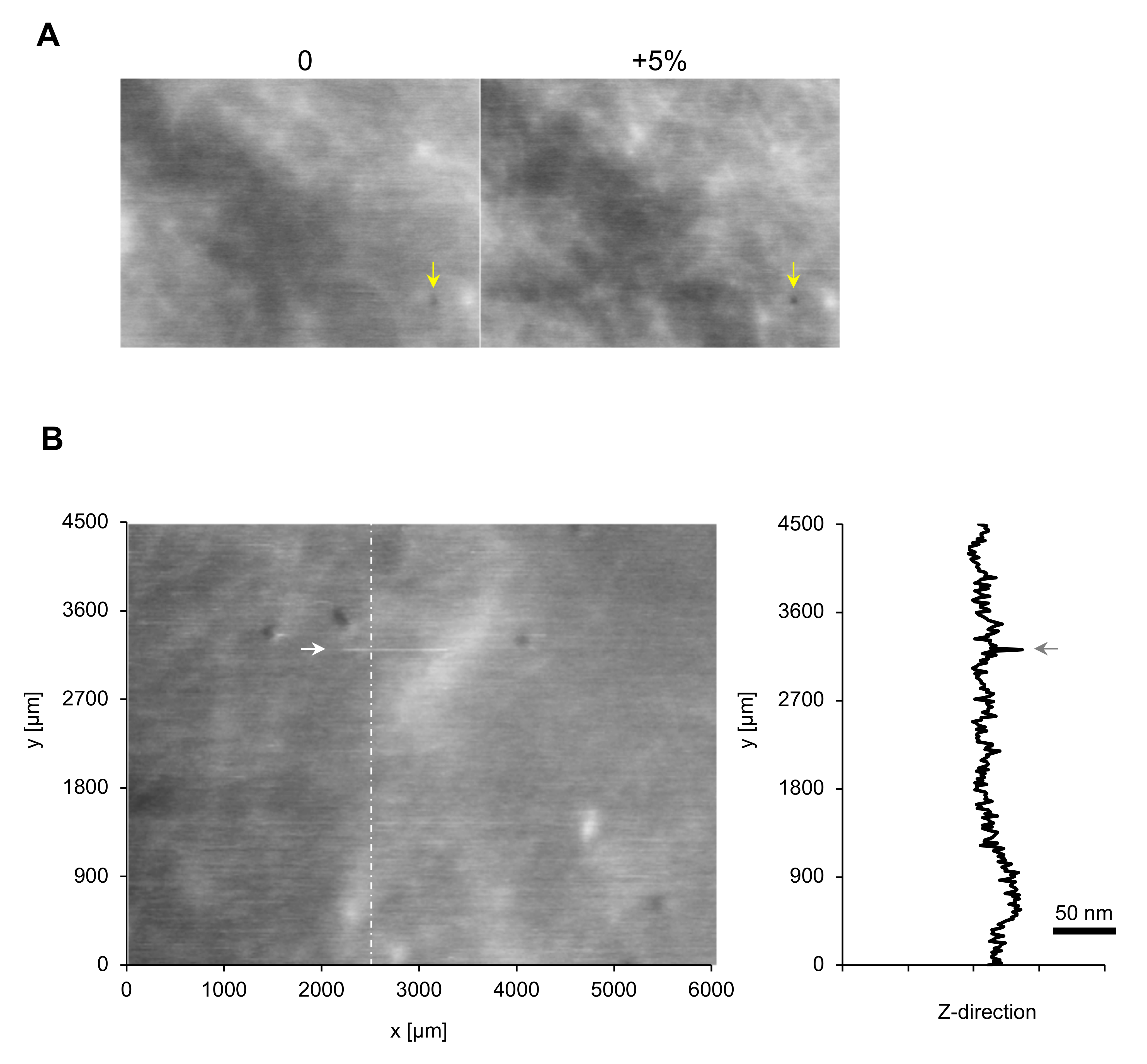

Supplement: S4 Fig — (A) Effect of a scanning parameter on the morphology of CCP. The time-lapse imaging of the CCP was conducted by changing the driving amplitude. As shown here, increasing the amplitude by 5% resulted in the image with clearer cytoskeletal pattern. In this case, the diameter of the CCP (arrow) changed by 9%. (B) An example of tip skipping. During the observation of the plasma membrane, tip skipping, which is indicated by an arrow in the left panel, occurs. The section profile (right panel) across the skipping region (dotted line in the left panel) indicates that it occurs at 1 or 2 consecutive lines and therefore does not affect morphological analyses of the CCP. Underlying data may be found in S1 Data. AFM, atomic force microscopy; CCP, clathrin-coated pit. (TIF) [file pbio.2004786.s004.tif]

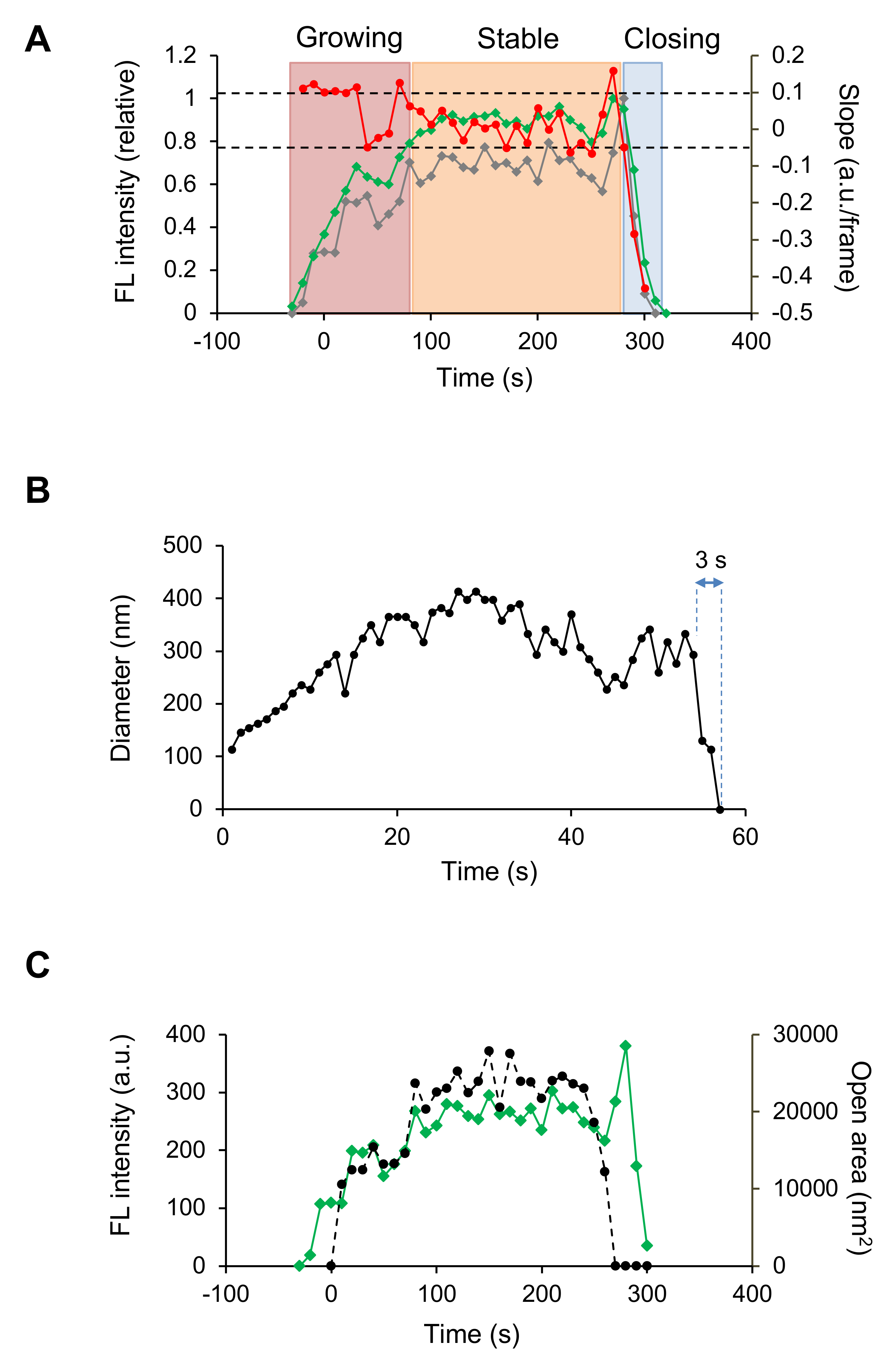

Supplement: S5 Fig — (A) Defining growing, stable, and closing phases. The fluorescence signal intensity of clathrin relative to the maximum value (grey) was first subjected to smoothing by 3 frame windows (green). The stable phase was defined when the slope of the averaged profile (red) was within −0.05 and +0.1 (/frame) for more than 4 frames. The growing and closing phases were defined as the period before and after the stable phase. (B) Analysis of closing step with a higher scanning rate at 1 s/frame (C) Time-lapse plot of the area of the pit. The pit that was analyzed in Fig 2B was re-analyzed to measure the pit area. The time-lapse AFM images were sequentially processed with Median Filter, Top Hat (Circle, Diameter: 20 pixel) and Open-Close filter (Circle, Diameter: 6 pixel) by MetaMorph software. Underlying data may be found in S1 Data. AFM, atomic force microscopy; CCP, clathrin-coated pit. (TIF) [file pbio.2004786.s005.tif]

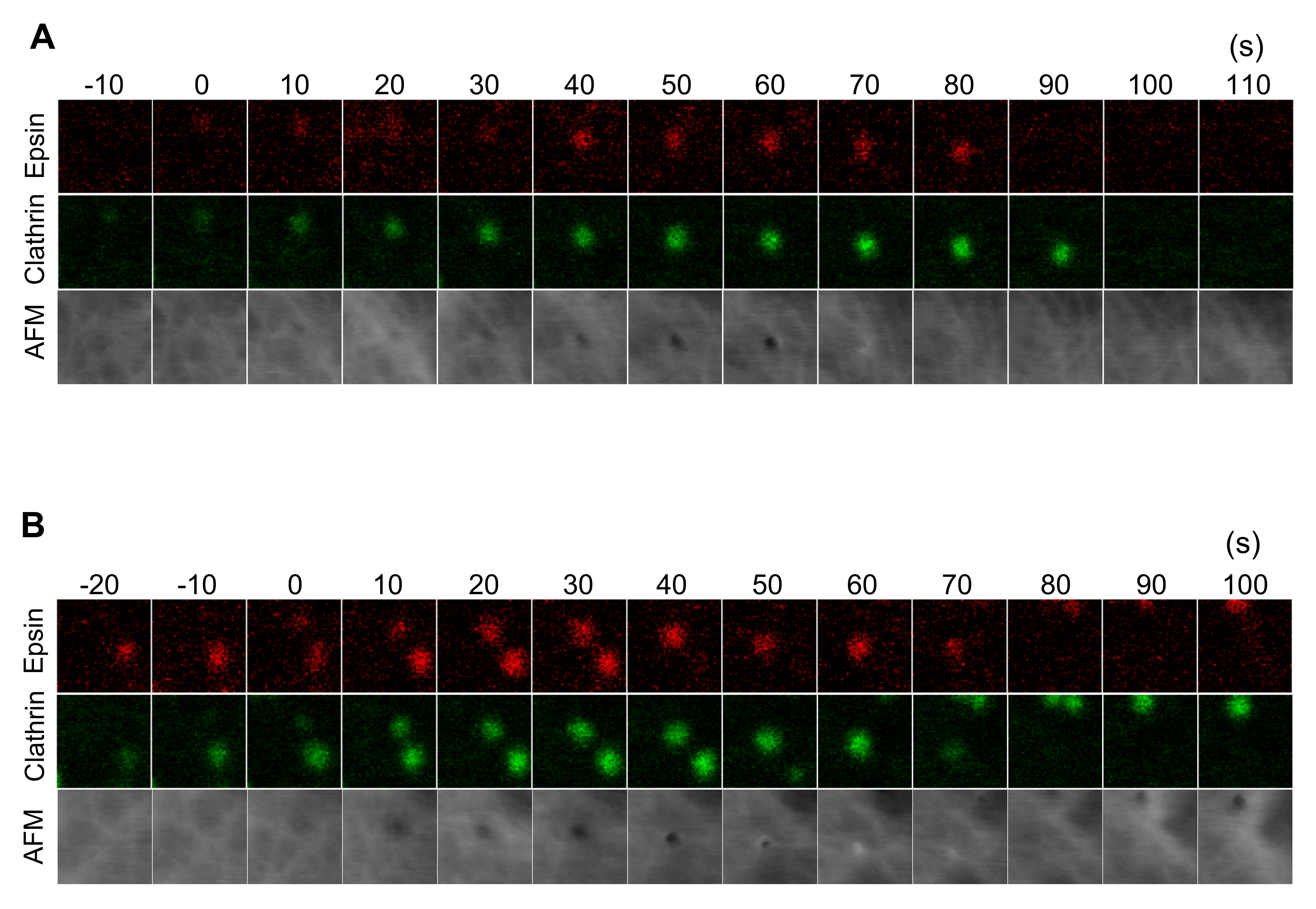

Supplement: S6 Fig — (A, B) Additional examples of hybrid time-lapse imaging of CCPs in a living COS-7 cell transiently co-expressing EGFP-CLCa and mCherry-epsin. Image size: 1.2 × 1.2 μm2. CCP, clathrin-coated pit; COS-7, CV-1 in origin with SV40 gene line 7; EGFP, enhanced green fluorescent protein; EGFP-CLCa, EGFP-fused clathrin light chain a. (TIF) [file pbio.2004786.s006.tif]

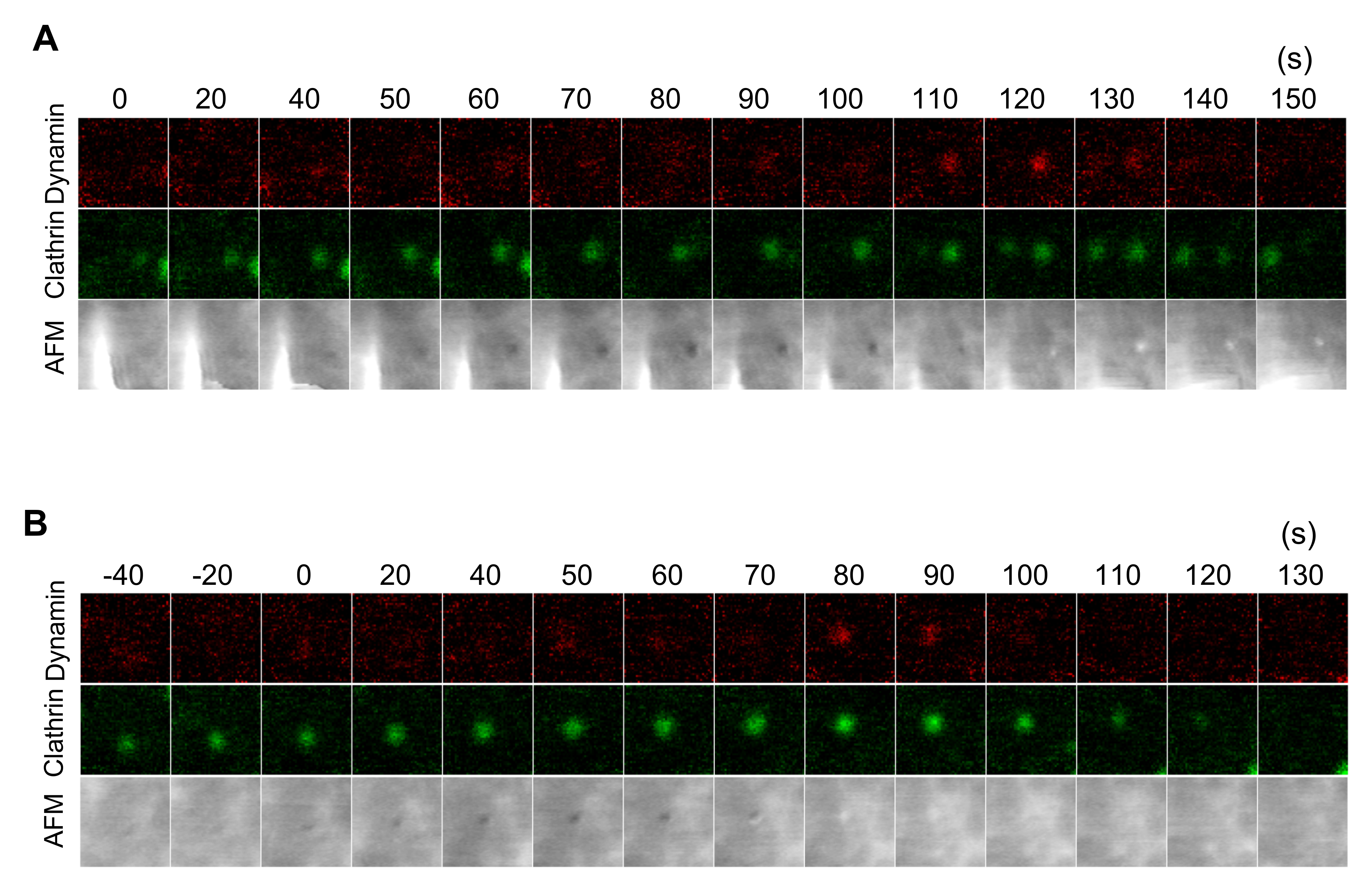

Supplement: S7 Fig — (A, B) Additional examples of hybrid time-lapse imaging of CCPs in a living COS-7 cell transiently co-expressing EGFP-CLCa and Dyn2-mCherry. Image size: 1.2 × 1.2 μm2. CCP, clathrin-coated pit; COS-7, CV-1 in origin with SV40 gene line 7; EGFP, enhanced green fluorescent protein; EGFP-CLCa, EGFP-fused clathrin light chain a. (TIF) [file pbio.2004786.s007.tif]

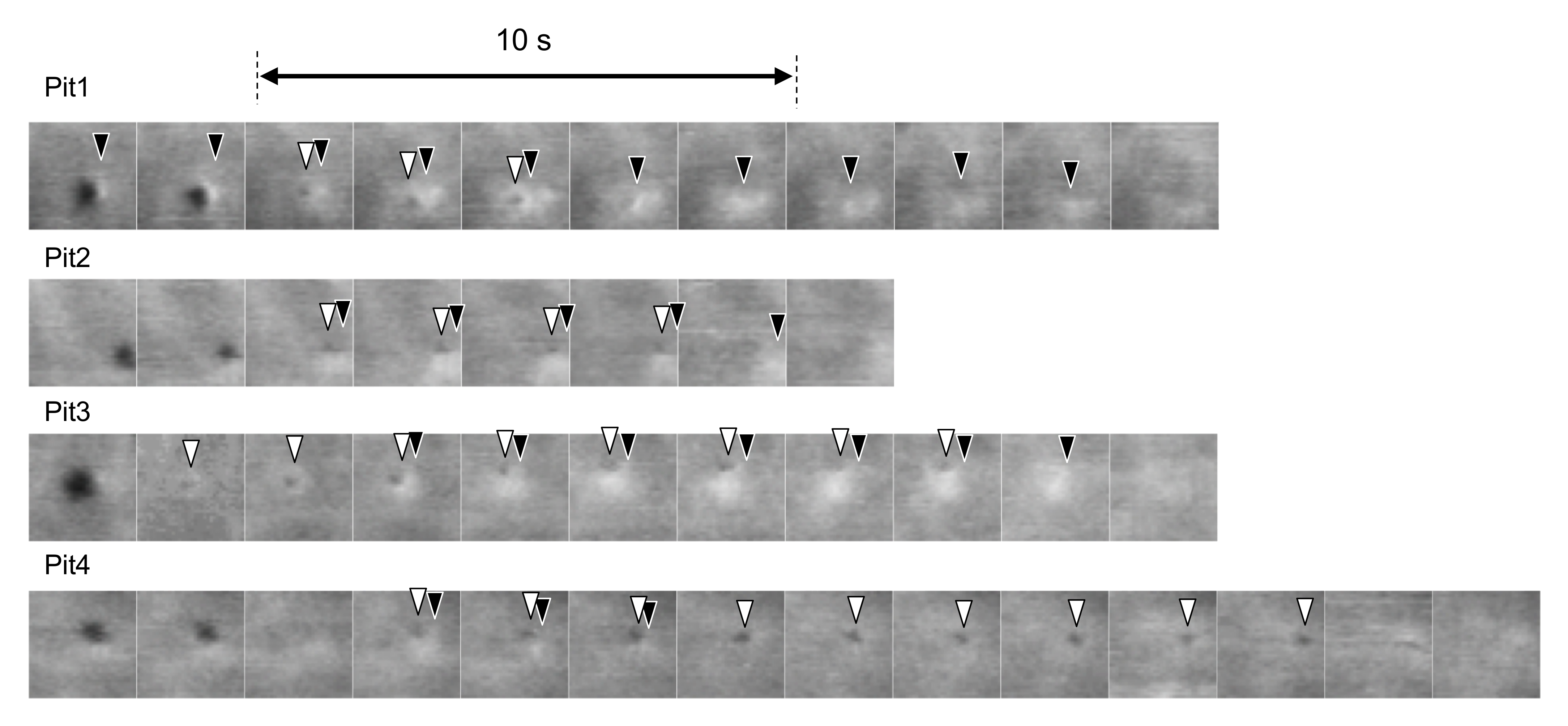

Supplement: S8 Fig — Sequential AFM images taken at 2-s intervals. Membrane swelling and small aperture are indicated by black and white arrowheads, respectively. Image size: 1.2 × 1.2 μm2. CCP, clathrin-coated pit. (TIF) [file pbio.2004786.s008.tif]

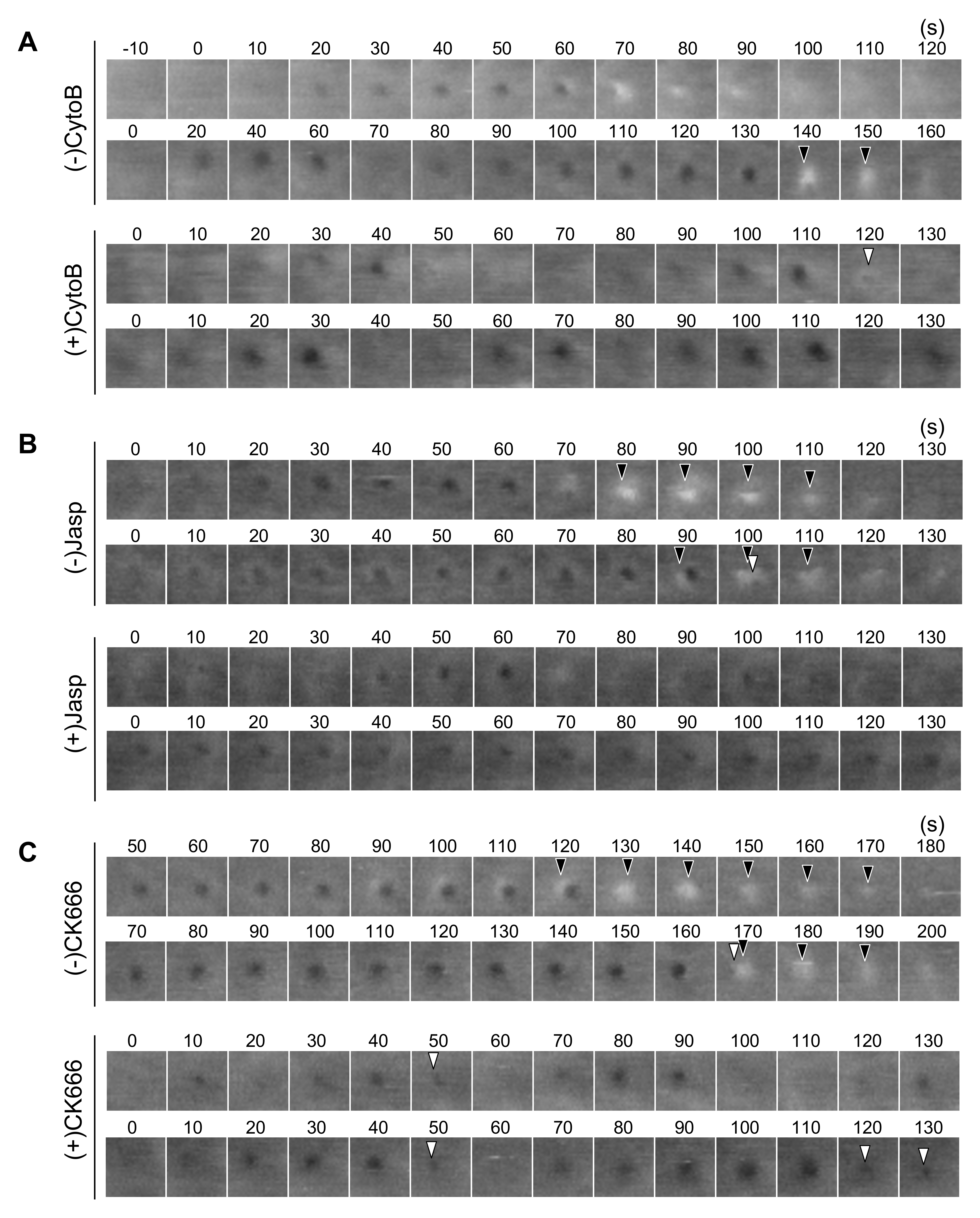

Supplement: S9 Fig — Time-lapse AFM images obtained in a living COS-7 cell before and after treatment with cytochalasin B, jasplakinolide, or CK666. Image size: 0.5 × 0.5 μm2. Capping and small apertures are indicated by black and white arrowheads, respectively. AFM, atomic force microscopy; CCP, clathrin-coated pit; COS-7, CV-1 in origin with SV40 gene line 7; cytoB, cytochalasin B; jasp, jasplakinolide. (TIF) [file pbio.2004786.s009.tif]

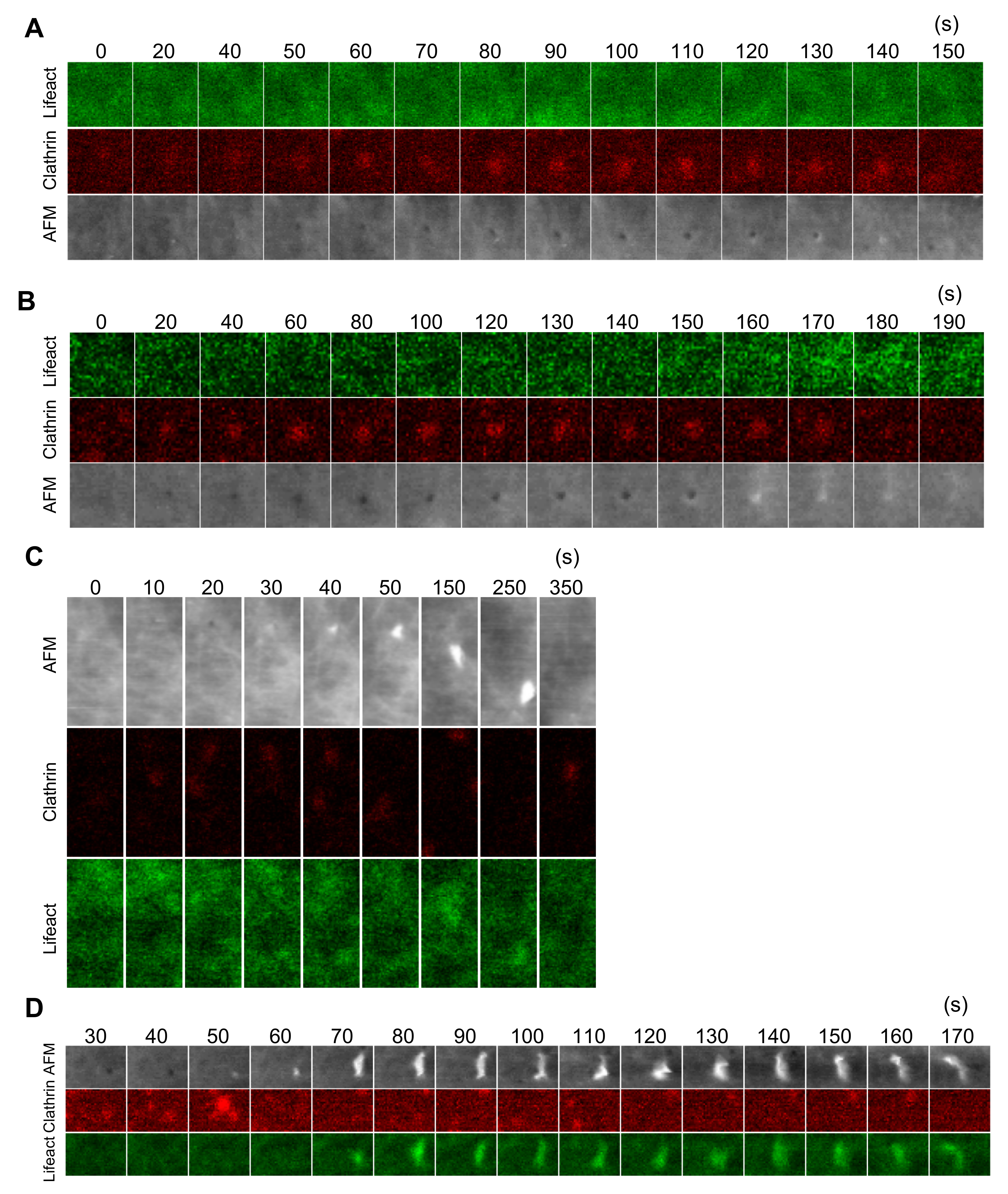

Supplement: S10 Fig — (A, B, C) Additional examples of hybrid time-lapse imaging of CCPs in a living COS-7 cell transiently co-expressing Lifeact-GFP and mCherry-CLCa. Image sizes in panels A and B: 1.2 × 1.2 μm2. Image size in panel C: 1.0 × 2.3 μm2. Image size in panel D: 1.74 × 1.23 μm2. In (C) and (D), a membrane swelling developed into a large ruffle-like protrusion. The actin signal was colocalized with such ruffle-like protrusions. CCP, clathrin-coated pit; CLCa, clathrin light chain a; COS-7, CV-1 in origin with SV40 gene line 7; GFP, green fluorescent protein. (TIF) [file pbio.2004786.s010.tif]
